# Automated Behavioral Workplace Intervention to Prevent Weight Gain and Improve Diet: The ChooseWell 365 Randomized Clinical Trial

**DOI:** 10.1001/jamanetworkopen.2021.12528

**Published:** 2021-06-07

**Authors:** Anne N. Thorndike, Jessica L. McCurley, Emily D. Gelsomin, Emma Anderson, Yuchiao Chang, Bianca Porneala, Charles Johnson, Eric B. Rimm, Douglas E. Levy

**Affiliations:** 1 Division of General Internal Medicine, Massachusetts General Hospital, Boston; 2 Harvard Medical School, Boston, Massachusetts; 3 Department of Nutrition and Food Services, Massachusetts General Hospital, Boston; 4 StudyMaker, Boston, Massachusetts; 5 Departments of Epidemiology and Nutrition, Harvard T.H. Chan School of Public Health, Boston, Massachusetts; 6 Channing Division of Network Medicine, Brigham and Women’s Hospital, Boston, Massachusetts; 7 Mongan Institute Health Policy Center, Massachusetts General Hospital, Boston

## Abstract

**Importance:**

Personalized interventions that leverage workplace data and environments could improve effectiveness, sustainability, and scalability of employee wellness programs.

**Objective:**

To test an automated behavioral intervention to prevent weight gain and improve diet using employee cafeteria purchasing data.

**Design, Setting, and Participants:**

This individual-level randomized clinical trial of a 12-month intervention with 12 months of follow-up was conducted among employees of a hospital in Boston, Massachusetts, who purchased food at on-site cafeterias that used traffic-light labels (ie, green indicates healthy; yellow, less healthy; red, unhealthy). Participants were enrolled September 2016 to February 2018. Data were analyzed from May to September 2020.

**Interventions:**

For 12 months, participants in the intervention group received 2 emails per week with feedback on previous cafeteria purchases and personalized health and lifestyle tips and 1 letter per month with peer comparisons and financial incentives for healthier purchases. Emails and letters were automatically generated using survey, health, and cafeteria data. Control group participants received 1 letter per month with general healthy lifestyle information.

**Main Outcomes and Measures:**

The main outcome was change in weight from baseline to 12 months and 24 months of follow-up. Secondary outcomes included changes in cafeteria purchases, including proportion of green- and red-labeled purchases and calories purchased per day, from baseline (12 months preintervention) to the intervention (months 1-12) and follow-up (months 13-24) periods. Baseline Healthy Eating Index-15 (HEI-15) scores were compared to HEI-15 scores at 6, 12, and 24 months.

**Results:**

Among 602 employees enrolled (mean [SD] age, 43.6 [12.2] years; 478 [79.4%] women), 299 were randomized to the intervention group and 303 were randomized to the control group. Baseline mean (SD) body mass index (BMI; calculated as weight in kilograms divided by height in meters squared) was 28.3 (6.6) and HEI-15 score was 60.4 (12.4). There were no between-group differences in weight change at 12 (0.2 [95% CI, −0.6 to 1.0] kg) or 24 (0.6 [95% CI, −0.3 to 1.4] kg) months. Compared with baseline, the intervention group increased green-labeled purchases by 7.3% (95% CI, 5.4% to 9.3%) and decreased red-labeled purchases by 3.9% (95% CI, −5.0% to −2.7%) and calories purchased per day by 49.5 (95% CI, −75.2 to −23.9) kcal more than the control group during the intervention period. In the intervention group, differences in changes in green (4.8% [95% CI, 2.9% to 6.8%]) and red purchases (−3.1% [95% CI, −4.3% to −2.0%]) were sustained at the 24-month follow-up. Differences in changes in HEI-15 scores were not significantly different in the intervention compared with the control group at 6 (2.2 [95% CI, 0 to 4.4]), 12 (1.8 [95% CI, −0.6 to 4.1]), and 24 (1.6, 95% CI, −0.7 to 3.8]) months.

**Conclusions and Relevance:**

The findings of this randomized clinical trial suggest that an automated behavioral intervention using workplace cafeteria data improved employees’ food choices but did not prevent weight gain over 2 years.

**Trial Registration:**

ClinicalTrials.gov Identifier: NCT02660086

## Key Points

**Question:**

Can an automated intervention using workplace cafeteria purchasing data to provide personalized feedback and nudges prevent weight gain and improve dietary choices?

**Findings:**

In this randomized clinical trial of 602 employees at a single hospital site, the 1-year automated intervention did not prevent weight gain. The intervention group significantly increased healthy cafeteria purchases during the 1-year intervention, and this was sustained during 1-year of additional follow-up.

**Meaning:**

These findings suggest that an automated intervention using food purchasing data improved workplace food choices over 2 years, but dietary changes did not prevent weight gain.

## Teaser

This randomized clinical trial tests the effectiveness of an automated behavioral intervention using employee cafeteria purchasing data to prevent weight gain and improve diet.

## Introduction

Obesity and unhealthy diet are leading risk factors for chronic disease and mortality.^1,2,3^ US adults gain a mean of 0.7 to 0.9 kg per year.^4,5^ With approximately 150 million US adults employed and spending half their waking hours working,^6^ the workplace provides an opportunity to promote health. Multiple workplace factors may contribute to unhealthy behaviors and weight gain, including psychosocial stressors, shift work, and sedentary jobs.^7^ Over the past 3 decades, most workplace wellness programs have used short-term education-based interventions that require employees to take time away from work and other responsibilities.^8,9,10^ Few provide long-term behavioral and environmental supports to sustain employees’ healthy choices. Personalized approaches that use insights from behavioral economics and leverage workplace data and environments could improve the effectiveness, sustainability, and scalability of workplace health promotion.

Despite widespread adoption of workplace wellness programs,^10^ few randomized clinical trials have demonstrated improvements in employees’ health outcomes or long-term health behaviors, such as diet.^8,11,12^ In a cluster-randomized trial of 160 worksites, an 8-module educational intervention with modest financial incentives implemented over 18 months increased self-report of health behaviors but did not improve health outcomes.^13^ In another randomized clinical trial of 4834 university employees, a 2-year comprehensive program, including biometric screenings, annual health risk assessments, and wellness classes, improved employees’ beliefs about their health but did not change measures of health or behaviors.^14^

Results of these trials suggest that strategies commonly used in workplace wellness programs, such as educational classes or biometric screenings, may not be effective for making lasting changes in health or behaviors. In workplace and nonworkplace settings, personalized interventions using feedback and behavioral nudges, such as peer comparisons and financial incentives, have demonstrated short-term success improving physical activity, food choices, and weight.^15,16,17,18,19^ There has been little research to evaluate the long-term effectiveness of personalized approaches on health outcomes and behaviors.

Previous studies have demonstrated that implementing a hospital workplace cafeteria traffic-light labeling and choice architecture (ie, product placement) intervention improved employees’ healthy food choices.^20,21,22^ In the same workplace, a pilot randomized study showed that peer comparisons and small financial incentives further increased employees’ healthy purchases.^19^ Building on these preliminary findings, the ChooseWell 365 study was a randomized clinical trial that tested a 12-month automated, personalized behavioral intervention to prevent weight gain and improve diet in hospital employees. The intervention was delivered remotely, integrated into the workday, and linked to the existing cafeteria traffic-light labeling system.^23^ Weight, diet, and health outcomes were evaluated over 24 months.

## Methods

The trial protocol (Supplement 1) for this randomized clinical trial was approved by the institutional review board at Mass General Brigham (formerly Partners Healthcare). All participants provided written informed consent. The trial was reported according to the Consolidated Standards of Reporting Trials (CONSORT) reporting guideline.

### Study Design

ChooseWell 365 was a randomized clinical trial of a behavioral intervention. Participants were enrolled between September 2016 and February 2018 and took part over 24 months, with a 12-month intervention period plus 12 months of follow-up. Participants provided written informed consent and were randomized to intervention or control groups after completing baseline measures. Surveys, dietary recalls, and assessments were completed at baseline and 6- (recalls only), 12-, and 24-month follow-ups. Staff conducting in-person assessments were blinded to assignment.

### Setting

This randomized clinical trial was conducted on the main campus of Massachusetts General Hospital (MGH), a large academic teaching hospital in Boston, Massachusetts. During the study, there were 7 on-site MGH food service locations, including 4 full-service, 1 grab-and-go, and 2 coffee shop cafeterias. No outside food service vendors were located on campus. All full-time hospital employees could opt to pay for cafeteria purchases with their employee identification (ID) card using payroll deduction.

The hospital cafeterias have used traffic-light labels since 2010.^20^ The traffic-light system was developed by MGH dietitians based on US Department of Agriculture guidelines^24^ and has been described in detail elsewhere.^20,21,23^ Briefly, red, yellow, and green labels were assigned to every food and beverage item based on an algorithm that factored in calories, saturated fat, and nutrient density. A green rating indicated the highest level of healthfulness, and red was the lowest. Examples of green items include black tea, fruit cup, sautéed spinach, and herb-baked chicken. Red items include chai tea latte, large muffins, french fries, and chicken tenders. Traffic-light labels were visible on menu boards, shelf labels, and directly on food packages prepared by cafeteria staff (eg, premade sandwiches).

### Participants

**Figure 1.**
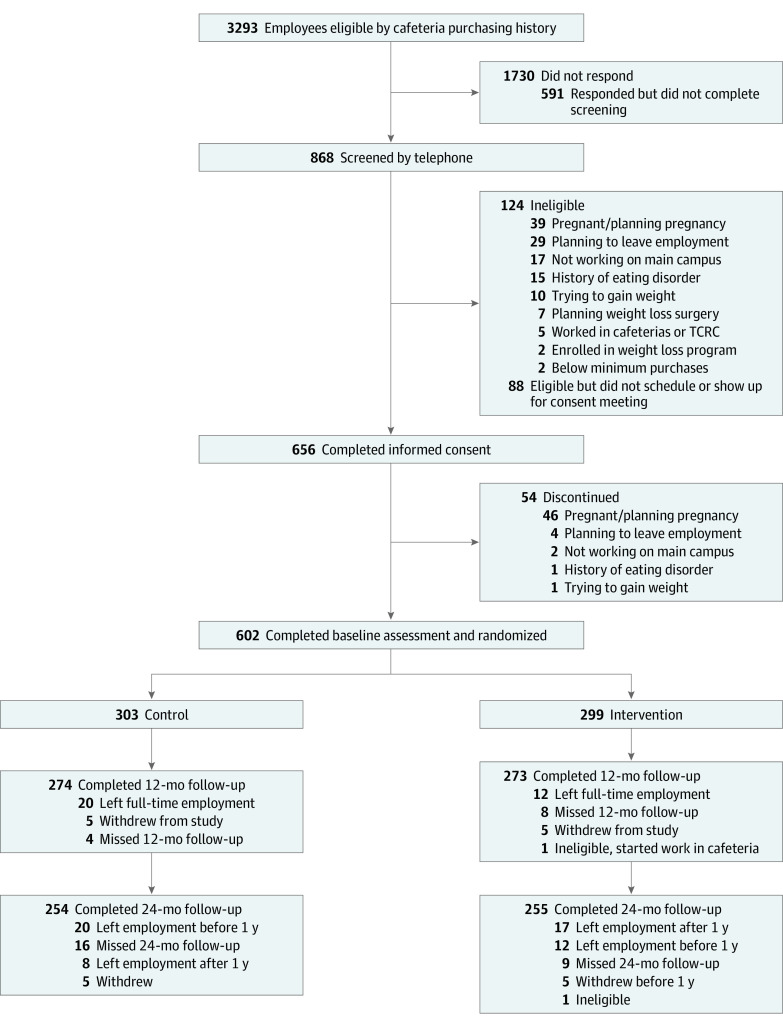
Study Recruitment Flowchart TCRC indicates Translational and Clinical Research Center.

Employees were potentially eligible for the study if they were between the ages of 20 and 75 years and used their ID for cafeteria purchases on the main campus at least 4 times a week for at least 6 weeks over a 12-week period prior to enrollment. Emails were sent to 3293 employees who fulfilled these criteria to invite them to participate (Figure 1). Employees who responded were screened by telephone for eligibility. Exclusion criteria were plans to leave employment in the next year (eg, retirement), current pregnancy, desire to gain weight, history of eating disorder, weight loss surgery in prior 6 months or planned in the upcoming year, current enrollment in a weight loss program, and working in the MGH cafeteria or in the Translational and Clinical Research Center (TCRC), where study visits took place. If eligible, the participant scheduled a consent meeting with study staff. All study communication was delivered by a participant’s work email and home postal service mail. Study staff provided instructions to all participants during the initial consent meeting about how to access work email remotely. Participants were emailed the baseline survey and instructions for completing dietary recalls electronically. A baseline assessment visit was scheduled at the TCRC for completion of weight measurements, resting energy expenditure (measured using VMAX Encore 29 metabolic cart [Viasys Healthcare, Carefusion]),^25^ International Physical Activity Questionnaire,^26^ and blood work. Demographic characteristics, including race/ethnicity, were self-reported on the baseline survey and were collected to examine whether they were associated with differences in outcomes. The baseline survey also collected data that were used to inform the personalized messages delivered to intervention group participants, including medical history, family history of cardiovascular disease or diabetes, eating and activity behaviors, sleep patterns, and weight history.

### Randomization

Participants were randomly assigned to the intervention or control group after completing the baseline survey and study visit (Figure 1). Randomization was stratified by the participant’s response to a survey question asking whether they wanted to lose weight or maintain their current weight in the upcoming year and used a computer-generated block randomization scheme with block sizes of 6. All randomized participants received a 10% discount on cafeteria purchases made with their ID card during the 24-month study. Participants were instructed that the ID card should only be used for items intended for personal consumption.

### Intervention

Development of the study intervention has been described in detail elsewhere.^23^ After the baseline visit, participants randomized to the intervention group were emailed a result letter that included their daily calorie budget, calculated using the measured resting energy expenditure and physical activity levels and accounted for a participant’s desire to lose or maintain weight. The letter also included fasting glucose, hemoglobin A_1c_ (HbA_1c_), and lipid profile results.

Participants received 2 emails per week that were automatically generated by the ChooseWell 365 software platform developed for this study.^23^ The first email, sent on Tuesdays, provided a log of all cafeteria items purchased during the prior week. The email included a colored summary graphic, as well as a list of daily items, calories, and remaining calories for each day (daily calorie goal minus total purchased calories) to provide a benchmark to guide future food choices. The second email, sent on Thursdays, provided 2 personalized tips about healthy eating, physical activity, or disease prevention, as well as a simple and healthy recipe. Prior to the study start, a database of more than 350 messages was developed by the study dietitian (E.D.G.) and physician (A.N.T.) using 6 predetermined domains: weight and energy balance, disease risk, workplace food, home food, barriers to healthy eating, and physical activity. The software platform pulled messages from the database that were triggered by participants’ weekly cafeteria purchases, baseline survey responses, and health measurements (eTable 1 in Supplement 2).

Participants received a monthly letter in the mail that included a graph illustrating the participant’s monthly proportion of green, yellow, and red cafeteria items purchased, compared with all MGH employees and with the healthiest MGH employees, defined as employees with 80% or more green purchases.^19,23^ The letter also provided a green goal to earn a financial incentive by increasing green purchases in the next month. A reward of $20 could be earned for passing 40%, 60%, or 80% monthly green purchase thresholds. Each threshold could only be rewarded once. If a participant maintained green purchases above a new threshold (but did not pass the next threshold), or if they maintained greater than 80% green, they earned $5 for the month. Employees with the least healthy purchasing at baseline (ie, <40% green) could earn the most money over 12 months (maximum $115).

### Control

Participants assigned to the control group received a letter by email after the baseline visit with blood test results (glucose, HbA_1c_, and lipids). During the 12-month intervention period, control participants did not receive any emails; they received a monthly letter with standard healthy lifestyle tips, such as the benefits of eating vegetables and exercising regularly. To ensure that the intervention group received the same standard lifestyle information as the control group, one of the intervention group emails each month provided the same message.

### Outcome Measures

The primary outcome was change in weight at 12 months compared with baseline. Secondary health outcomes included changes in systolic and diastolic blood pressure (BP); total, low-density lipoprotein, and high-density lipoprotein cholesterol; triglycerides; HbA_1c_; body mass index (BMI; calculated as weight in kilograms divided by height in meters squared); and waist circumference at 12 and 24 months. All health measures were measured at study visits by nurses in the TCRC. If a participant was unable to attend a visit, research staff offered to meet them to measure weight; 13 participants at 12 months and 15 participants at 24 months had weight measured this way.

Cafeteria purchase outcomes were changes in the percentages of green-, yellow-, and red-labeled workplace food purchased, the workplace healthy purchasing score, and calories purchased per day during the intervention (months 1-12) and follow-up (months 13-24) periods compared with the baseline period (12 months preintervention). Most participants (519 participants [86.2%]) had 12 months of baseline purchasing data; all participants had at least 4 months of baseline purchasing data. The previously validated healthy purchasing score was derived by weighting purchases of red items to be 0, yellow to be 0.5, and green to be 1 and was converted to percentage by multiplying the score by 100 (range, 0%-100% healthy).^27^ Mean calories purchased per day was calculated for days that participants made purchases.

Dietary intake outcomes were changes from baseline in Healthy Eating Index-15 (HEI-15)^28^ scores at 6, 12, and 24 months. The HEI-15 was calculated based on 2 Automated Self-Administered 24-hour (ASA24) dietary recalls^29^ completed at baseline and each follow-up time point. ASA24 is a free web-based tool for dietary intake assessment developed by the National Cancer Institute.^30^ If a participant did not complete a second ASA24, the HEI-15 was calculated based on 1 ASA24.^30^ The HEI-15 measures overall dietary quality, consistent with US Department of Agriculture guidelines, and scores range from 0 (least healthy) to 100 (healthiest). The mean score for US adults is 59.^31^ The number of ASA24s completed at each time point was similar in both groups (eTable 2 in Supplement 2).

Intervention participants completed survey questions at 12 months about perceived effectiveness of the 6 intervention components: cafeteria traffic light labels, cafeteria purchasing feedback, personalized health tips, healthy recipes, peer comparison feedback, and financial incentives. Participants were asked to indicate all components that they considered helpful for making healthier choices.

### Statistical Analysis

We conducted an intent-to-treat analysis using a difference-in-differences approach to compare changes in weight, BMI, waist circumference, blood pressure, cholesterol, HbA_1c_, cafeteria purchases (over 12-month periods), and HEI-15 scores between the intervention and the control groups. All missing data from participants who left employment, were pregnant, or missed the follow-up assessment for any other reason (Figure 1) were imputed at 6- (for HEI-15 only), 12-, and 24-month follow-ups using multiple imputation that used information from baseline covariates, prior data, and all available outcomes from the same time point. Mixed-effect models were used to account for the repeated measures data structure. The estimates from 5 sets of imputed data were combined in the final results. Monthly cafeteria purchases, which were fully observed using administrative data, were also examined for both groups over 24 months using nonimputed data to observe proportions of purchases that were green, yellow, and red over time. To explore intervention effectiveness, post hoc analyses using nonimputed data were conducted in 3 subgroups: baseline weight goal (loss vs maintenance), baseline BMI (<25, 25-29.9, and ≥30), and tertile of baseline metabolic equivalent (MET)–minutes of physical activity per week.^26^ All analyses were conducted using SAS statistical software version 9.4 (SAS Institute).

The target enrollment for this study was based on an a priori calculation that demonstrated a sample size of 600 participants would ensure 90% power to detect a difference of 0.5 kg weight change between groups at 12 months with a 2-sided significance level of *P* = .05, accounting for 10% loss to follow-up. Data were analyzed from May to September 2020.

## Results

A total of 602 hospital employees completed baseline data collection and were randomized (Figure 1). Overall, the mean (SD) age was 43.6 (12.2) years, and 478 (79.4%) were women. A total of 488 participants (81.1%) were White, 54 participants (9.0%) were Black, 27 participants (4.5%) were Asian, and 34 participants (5.6%) were Hispanic. The baseline mean (SD) BMI was 28.3 (6.6) and HEI-15 score was 60.4 (12.4). More participants (503 participants [83.6%]) had a goal of weight loss than weight maintenance (99 participants [16.4%]). A total of 299 participants were randomized to the intervention group, and 303 participants were randomized to the control group. Baseline characteristics were balanced in the study groups (Table 1).

**Table 1.** Participant Characteristics at Baseline

Characteristic	No. (%)	
	Intervention group (n = 299)	Control group (n = 303)
Age, mean (SD), y	43.5 (12.0)	43.8 (12.5)
Sex		
Men	69 (23.1)	55 (18.2)
Women	230 (76.9)	248 (81.8)
Race		
White	246 (82.3)	242 (79.9)
Black	28 (9.4)	26 (8.6)
Asian	14 (4.7)	13 (4.3)
Other or not reported^a^	11 (3.7)	22 (7.3)
Hispanic ethnicity	17 (5.7)	17 (5.6)
Education		
High school or some college	36 (12.0)	39 (12.9)
College degree	123 (41.1)	117 (38.6)
Graduate degree	138 (46.2)	146 (48.2)
Not reported	2 (0.7)	1 (0.3)
Job category		
Administrative or service	36 (12.0)	48 (15.8)
Crafts or technicians	36 (12.0)	31 (10.2)
Management or professionals	193 (64.5)	184 (60.7)
Physicians or PhDs	34 (11.4)	40 (13.2)
Weight category		
Normal (BMI <25)	109 (36.5)	119 (39.3)
Overweight (BMI 25-29.9)	92 (30.8)	100 (33.0)
Obese (BMI ≥30)	98 (32.8)	84 (27.7)
Hypertension	51 (17.1)	49 (16.2)
Hyperlipidemia	51 (17.1)	60 (19.8)
Prediabetes or diabetes	20 (6.7)	25 (8.3)
Current smoker	8 (2.7)	9 (3.0)
Physical activity, median (IQR), MET-min/wk	4077 (2075-9213)	3953 (1977-7728)
Healthy Eating Index score, mean (SD)^b^	59.7 (12.3)	61.0 (12.5)
Weight goal		
Lose weight	248 (82.9)	255 (84.2)
Maintain weight	51 (17.1)	48 (15.8)

Abbreviations: BMI, body mass index (calculated as weight in kilograms divided by height in meters squared); IQR, interquartile range; MET, metabolic equivalent of task.

^a^ Includes Native Hawaiian or Pacific Islander, more than 1 race, or prefer not to answer.

^b^ Range, 0 to 100, with higher score indicating healthier eating.

### Weight and Cardiometabolic Outcomes

At 12 months, there was no difference in change in weight between intervention and control groups (mean difference, 0.2 [95% CI, −0.6 to 1.0] kg) (Table 2). Weight change at the 24-month follow-up was also not different. All secondary health outcomes were not significantly different between groups (Table 2).

**Table 2.** Changes in Health Measures and Workplace Cafeteria Purchases

Measure	Mean (95% CI)		Difference in changes, intervention-control, mean (95% CI)	*P* value
	Intervention group	Control group		
**Health **				
Weight, kg				
Baseline, mean (SD)	79.8 (18.8)	77.0 (18.3)	NA	NA
Change				
12-mo	0.6 (0.1 to 1.1)	0.4 (−0.1 to 0.9)	0.2 (−0.6 to 1.0)	.70
24-mo	1.5 (0.7 to 2.2)	0.9 (0.2 to 1.6)	0.6 (−0.3 to 1.4)	.20
BMI				
Baseline, mean (SD)	28.6 (6.6)	28.0 (6.5)	NA	NA
Change				
12-mo	0.2 (0.1 to 0.4)	0.2 (0 to 0.4)	0.1 (−0.2 to 0.3)	.72
24-mo	0.5 (0.3 to 0.8)	0.4 (0.1 to 0.6)	0.2 (−0.1 to 0.5)	.25
Waist, cm				
Baseline, mean (SD)	95.6 (17.8)	93.4 (16.2)	NA	NA
Change				
12-mo	1.0 (−0.3 to 2.4)	1.3 (0.5 to 2.2)	−0.3 (−1.8 to 1.3)	.72
24-mo	1.3 (0 to 2.7)	1.8 (0.9 to 2.6)	−0.4 (−2.0 to 1.1)	.57
Systolic BP, mm Hg				
Baseline, mean (SD)	121.4 (14.4)	120.4 (14.7)	NA	NA
Change				
12-mo	−1.9 (−3.4 to −0.3)	−0.5 (−2.4 to 1.4)	−1.3 (−3.6 to 0.9)	.24
24-mo	−0.2 (−1.9 to 1.6)	−1.7 (−3.4 to 0.1)	1.5 (−0.7 to 3.7)	.19
Diastolic BP, mm Hg				
Baseline, mean (SD)	71.3 (9.9)	70.2 (9.9)	NA	NA
Change				
12-mo	−2.4 (−3.5 to −1.3)	−0.8 (−2.1 to 0.4)	−1.6 (−3.2 to 0.1)	.07
24-mo	−1.7 (−2.8 to −0.6)	−1.8 (−3.0 to −0.6)	0.1 (−1.5 to 1.6)	.94
Total cholesterol, mg/dL				
Baseline, mean (SD)	183.8 (34.8)	182.7 (38.2)	NA	NA
Change				
12-mo	−0.3 (−3.2 to 2.6)	1.0 (−2.5 to 4.6)	−1.3 (−5.6 to 2.9)	.54
24-mo	2.5 (−0.7 to 5.6)	0.9 (−3.3 to 5.0)	1.6 (−3.5 to 6.7)	.53
LDL cholesterol, mg/dL				
Baseline, mean (SD)	102.1 (30.6)	101.5 (33.2)	NA	NA
Change				
12-mo	0.8 (−1.9 to 3.4)	2.0 (−0.7 to 4.6)	−1.2 (−4.8 to 2.4)	.51
24-mo	4.9 (1.9 to 7.9)	3.8 (0.3 to 7.2)	1.2 (−3.4 to 5.7)	.60
HDL cholesterol, mg/dL				
Baseline, mean (SD)	63.0 (18.5)	62.1 (17.4)	NA	NA
Change				
12-mo	−1.5 (−2.6 to −0.4)	−1.3 (−2.9 to 0.4)	−0.2 (−1.9 to 1.5)	.80
24-mo	−2.8 (−4.1 to −1.5)	−2.5 (−3.9 to −1.1)	−0.3 (−1.9 to 1.3)	.72
Triglycerides, mg/dL				
Baseline, mean (SD)	94.5 (56.3)	94.5 (67.0)	NA	NA
Change				
12-mo	−1.1 (−6.3 to 4.0)	1.8 (−5.7 to 9.2)	−2.9 (−10.8 to 5.1)	.48
24-mo	1.9 (−2.8 to 6.5)	−2.2 (−7.5 to 3.2)	4.0 (−3.4 to 11.5)	.29
Hemoglobin A_1c_, %				
Baseline, mean (SD)	5.5 (0.5)	5.5 (0.6)	NA	NA
Change				
12-mo	−0.1 (−0.1 to 0)	−0.1 (−0.2 to 0)	0 (−0.1 to 0.1)	.62
24-mo	−0.1 (−0.1 to 0)	−0.1 (−0.2 to −0.1)	0 (0 to 0.1)	.40
**Workplace food purchases**				
Green-labeled items, %				
Baseline, mean (SD)	50.1 (16.3)	52.3 (17.1)	NA	NA
Change				
12-mo^a^	9.4 (8.0 to 10.7)	2.0 (0.9 to 3.1)	7.3 (5.4 to 9.3)	<.001
24-mo^b^	5.7 (4.1 to 7.4)	0.9 (−0.6 to 2.4)	4.8 (2.9 to 6.8)	<.001
Yellow-labeled items, %				
Baseline, mean (SD)	34.2 (11.8)	33.1 (11.9)	NA	NA
Change				
12-mo^a^	−4.6 (−5.7 to −3.6)	−1.1 (−2.1 to −0.2)	−3.5 (−5.1 to −1.9)	<.001
24-mo^b^	−1.6 (−3.1 to −0.2)	0 (−1.3 to 1.4)	−1.7 (−3.4 to 0)	.05
Red-labeled items, %				
Baseline, mean (SD)	15.7 (10.8)	14.6 (10.9)	NA	NA
Change				
12-mo^a^	−4.8 (−5.5 to −4.0)	−0.9 (−1.6 to −0.2)	−3.9 (−5.0 to −2.7)	<.001
24-mo^b^	−4.1 (−5.0 to −3.2)	−1.0 (−2.0 to 0.1)	−3.1 (−4.3 to −2.0)	<.001
Healthy purchasing score, %				
Baseline, mean (SD)	67.2 (12.5)	68.8 (13.1)	NA	NA
Change				
12-mo^a^	7.1 (6.1 to 8.0)	1.5 (0.7 to 2.2)	5.6 (4.2 to 7.0)	<.001
24-mo^b^	4.9 (3.8 to 6.0)	0.9 (−0.2 to 2.0)	4.0 (2.6 to 5.3)	<.001
Calories purchased/d, kcal				
Baseline, mean (SD)	644.8 (252.4)	623.6 (268.7)	NA	NA
Change				
12-mo	−55.8 (−71.7 to −39.9)	−6.3 (−20.3 to 7.8)	−49.5 (−75.2 to −23.9)	<.001
24-mo	−60.9 (−83.6 to −38.3)	−44.4 (−66.1 to −22.5)	−16.6 (−42.5 to 9.3)	.21

Abbreviations: BMI, body mass index (calculated as weight in kilograms divided by height in meters squared); BP, blood pressure; LDL, low-density lipoprotein; HDL, high-density lipoprotein; NA, not applicable.

SI conversion factors: To convert cholesterol to millimoles per liter, multiply by 0.0259; triglycerides to millimoles per liter, multiply by 0.0113; hemoglobin A_1c_ to proportion of total hemoglobin, multiply by 0.01.

^a^ Compares purchases during 12-month intervention period (months 1 to 12) to purchases during 12-month baseline period (12 months preintervention).

^b^ Compares purchases during 12-month follow-up period (months 13 to 24) to purchases during 12-month baseline period (12 months preintervention).

### Dietary Measures

**Figure 2.**
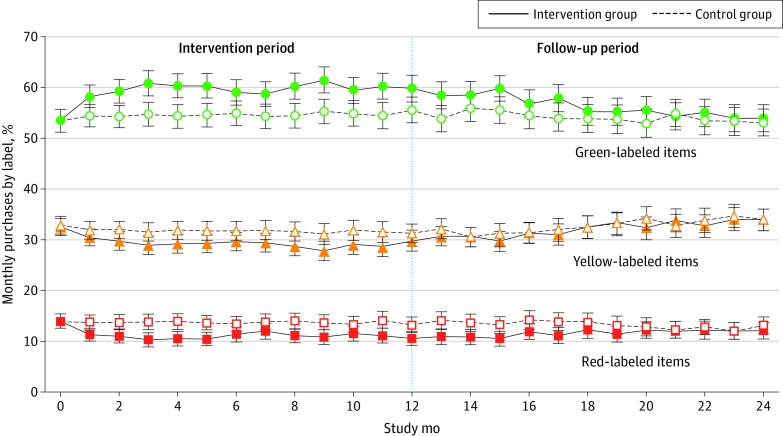
Monthly Proportion of Red, Yellow, and Green Cafeteria Purchases Over 2 Years Error bars indicate 95% CIs.

**Figure 3.**
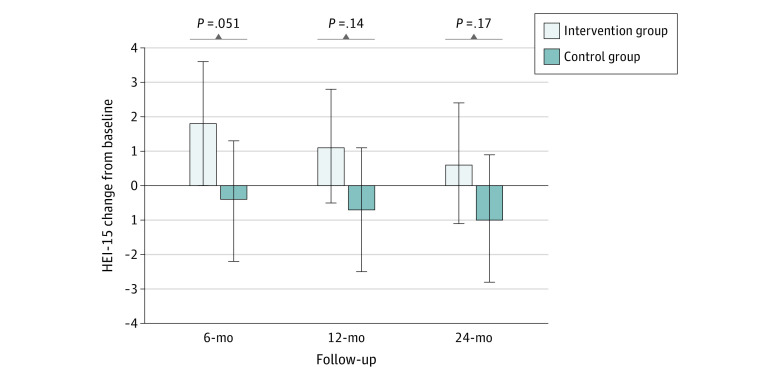
Changes From Baseline Healthy Eating Index-15 (HEI-15) Scores at Follow-up Error bars indicate 95% CIs.

Comparing the intervention period (months 1 to 12) with the baseline period (12 months preintervention), the intervention group increased purchases of green-labeled items by 7.3 (95% CI, 5.4 to 9.3) percentage points and decreased purchases of red-labeled items by 3.9 (95% CI, −5.0 to −2.7) percentage points more than the control group (Table 2). The intervention group also increased their healthy purchasing score by 5.6 (95% CI, 4.2 to 7.0) percentage points and decreased calories purchased per day by 49.5 (95% CI, −75.2 to −23.9) kcal compared with the control group. Differences in green and red purchases and healthy purchasing scores remained significant during the follow-up period (months 13 to 24) compared with the baseline period. Figure 2 shows monthly red, yellow, and green cafeteria purchases over the 24-month study. There were no significant differences in changes in HEI-15 scores between the intervention and control groups at 6 months (2.2 [95% CI, 0 to 4.4]), 12 months (1.8 [95% CI, −0.6 to 4.1]), or 24 months (1.6 [95% CI, −0.7 to 3.8]), (Figure 3).

### Exploratory Subgroup Analyses

Subgroup analyses of weight, BMI, cafeteria purchasing, and HEI-15 score outcomes at 12 and 24 months are displayed in eTables 3, 4, and 5 in Supplement 2. Overall, differences in intervention vs control group changes in weight, BMI, and the healthy purchasing score at 12 and 24 months did not differ by subgroup. The HEI-15 increased significantly at 12 months for intervention vs control participants with a goal of weight loss and participants with baseline BMI 30 or greater (eTable 3 in Supplement 2).

### Perceived Effectiveness of Intervention

At 12 months, 268 intervention participants (90%) completed survey questions about the intervention. Among these, 246 participants (92%) stated at least 1 of the 6 components had helped them make healthier choices (eFigure 1 in Supplement 2). Traffic-light labels were the most frequently cited helpful item (213 participants [79.5%]). Most participants rated 2 or more components as helpful (eFigure 2 in Supplement 2).

## Discussion

In this randomized clinical trial, a 12-month intervention using workplace cafeteria purchasing data for personalized feedback and behavioral nudges improved employees’ workplace food choices but did not prevent weight gain over 2 years. This low-touch intervention was delivered automatically and remotely, without requiring time-intensive classes or counseling. The intervention strategy was effective for sustaining healthier dietary behaviors, but dietary changes were not large enough to change cardiometabolic outcomes within the 2-year period.

Weight gain prevention is a population-based strategy that can be applied to almost all adults,^32,33^ but few workplace-based trials have tested this approach. Weight gain prevention studies have had mixed results because of short duration and heterogeneity of interventions.^34,35,36^ A 2016 randomized clinical trial^37^ of 250 adults aged 18 to 35 years who were overweight demonstrated that a 12-week intervention with 5 coaching calls plus text messages and emails resulted in less weight gain at 9 months follow-up. The Study of Novel Approaches to Weight Gain Prevention randomized clinical trial^38^ of 599 adults aged 18 to 35 years tested 2 interventions that included 10 face-to-face meetings: one focused on large changes (ie, losing 2.3 to 4.5 kg) and one on focused small changes (ie, reducing 100 calories per day). Compared with the control, both intervention groups gained less weight over 3 years. The ChooseWell 365 intervention achieved a reduction of 50 calories per day but did not reach the daily 100-calorie deficit in the small changes group of the Study of Novel Approaches to Weight Gain Prevention trial.^38^ In the future, adding a physical activity tracking and feedback component to the ChooseWell 365 intervention could help close this calorie gap without adding resource-intensive counseling.

Few workplace interventions have achieved long-term improvements in employees’ diets. One 18-week study^39^ that randomized 291 employees with overweight, obesity, and/or diabetes to a low-fat vegan diet and weekly group meetings demonstrated reductions in saturated fat intake, weight, and lipids; however, one-third of the intervention group did not complete the study. ChooseWell 365 enrolled employees with no health-related inclusion criteria and was delivered remotely over the course of 1 year, without relying on self-reported intake and food logs, which are subject to reporting bias and are burdensome.^40,41^ While several trials have tested interventions using objective physical activity feedback and behavioral nudges,^15,16,17^ few have used objective dietary feedback and nudges. One randomized clinical trial of 274 young adults by Kerr et al^42^ tested tailored feedback based on a 4-day mobile food record using images of participants’ food and beverages but found no differences in diet at 6 months. In ChooseWell 365, it is likely that the 12 months of personalized feedback increased employees’ awareness of the cafeteria traffic-light labels and overall nutrition knowledge. However, the intervention did not result in statistically significant improvement of HEI-15 scores over 24-months.

### Limitations

This study has limitations. All participants worked at the same institution and purchased food in the same cafeterias that had implemented traffic-light labeling since 2010.^20,21^ Despite potential for contamination, purchasing patterns were significantly different between the intervention and control groups during the 2-year study. Although employees who participated in the study may have been more motivated to make healthy changes than nonparticipants, study participants’ baseline healthy cafeteria purchases were similar to the overall population.^21^ Additionally employees who remained employed but missed follow-ups may have gained more weight than those who completed all follow-ups. However, missed visits were infrequent in both groups. Only employees who were regular customers of the cafeterias were eligible to participate and may not be representative of all hospital employees. It is also worth noting that there were more women than men study participants, owing in part to the larger proportion of women employees at the hospital. Additionally, this study was conducted at a single urban hospital and may not be generalizable to other nonhospital workplaces.

## Conclusions

The findings of the ChooseWell 365 randomized clinical trial demonstrated that an automated intervention using food purchasing data improved employees’ healthy food choices over 2 years but did not prevent weight gain. In the future, the ChooseWell 365 platform could integrate other types of personalized data, such as accelerometry and genetics,^43^ and be delivered over longer periods of time to prevent weight gain and diet-related adverse health outcomes that develop over several years. ChooseWell 365 represents a new generation of workplace wellness programs that can leverage novel sources of data, be delivered remotely, and be scaled to large employee populations.

## Notes

Supplement 1. Trial Protocol

Supplement 2. **eTable 1.** Examples of Personalized Messages Emailed to Intervention Group **eTable 2.** Number of ASA24 Dietary Recalls Completed by Participants **eTable 3.** Subgroup Analysis 1: Changes in Weight and Dietary Measures by Weight Loss Goals **eTable 4.** Subgroup Analysis 2: Changes in Weight and Dietary Measures by Baseline BMI Category **eTable 5.** Subgroup Analysis 3: Changes in Weight and Dietary Measures by International Physical Activity Questionnaire Score Tertile **eFigure 1.** Intervention Components Rated Helpful for Making Healthier Choices by Intervention Participants **eFigure 2.** Total Number of Intervention Components Rated Helpful by Intervention Participants Who Rated 1 or More Components

Supplement 3. Data Sharing Statement
