# Supplementary material for: Automated Behavioral Workplace Intervention to Prevent Weight Gain and Improve Diet: The ChooseWell 365 Randomized Clinical Trial
Source: JAMA Netw Open. 2021 Jun 7;4(6):e2112528. doi: 10.1001/jamanetworkopen.2021.12528 (PMC8185595; doi:10.1001/jamanetworkopen.2021.12528)
Supplement: Supplement 1. — Trial Protocol [file jamanetwopen-e2112528-s001.pdf]

Detailed Protocol

I. Background and Significance

A. Interventions to prevent weight gain at the population level are needed to reverse the rising prevalence of obesity. Obesity is a major health concern in the United States and worldwide.[1,2] There is strong evidence that poor diet quality and increased energy intake are largely responsible for the rapid rise in obesity,[3,4] and U.S. adults gain an average of 1-2 pounds per year.[5] Although individual-level interventions can result in large weight changes among small groups of individuals, preventing obesity at the population level is going to require more permanent changes in physical, social, and cultural environments that will promote consumption of healthy, lower calorie foods and discourage unhealthy, energy-dense foods.[4,6,7] Preventing the chronic gradual increase in weight by making small changes to reduce energy intake has potential for a large impact on public health.[8] Recent studies have demonstrated that weight gain prevention interventions using environmental or individually tailored strategies can be effective in worksite, primary care, and community settings.[9-12]

B. The worksite is ideal for delivering population-based strategies to promote healthy behaviors. A worksite provides the opportunity to address employees' behaviors in their physical and social environments.[13,14] Most adults spend half of their waking hours at work, and the workplace has already established channels of communication, support networks, and opportunities for developing social and corporate norms.[13] With provisions in the Affordable Care Act encouraging worksite wellness, it is likely that more employers will be offering programs in the future.[13] Most worksite wellness programs are brief interventions that result in short-term changes in health or behavior (i.e. weight loss, getting a mammogram, completing a health risk assessment),[15,16] but the long-term effectiveness of programs has not been well-studied.[17-19] Worksite exercise and nutrition programs are expensive due to costs for staffing, providing access to exercise equipment and training, and accounting for time spent away from the job. However, cost-effectiveness analyses of worksite wellness programs suggest cost savings.[20,21]

C. Changing the food environment improves healthy choices of a population. The "food environment" is defined as the food and beverages that are included in the surroundings of an individual or a population (i.e. the worksite) and that impact the individual's or population's ability to make healthy choices. Several studies suggest that a healthy worksite food environment can have positive effects on attitudes toward eating a healthy diet and self-reported dietary intake.[22-25] Our research team designed and evaluated the long-term effectiveness of a traffic light labeling and product placement ("choice architecture") intervention in promoting healthier food and beverage purchases in the main cafeteria at MGH over two years.[26-29] There are now several other large employers that have adopted traffic-light labeling in their cafeterias, including Google, National Public Radio, Fidelity Investments, and Brigham and Women's Hospital. Although demonstrating improvements in healthy food choices at work is an important first step, the critical next phase of this research is to determine if worksite food environment interventions can prevent weight gain and improve the health of a population of workers.

D. Providing personalized feedback can improve healthy food choices and reduce weight. Studies of tailored dietary feedback delivered electronically or in print have demonstrated short-term changes in fruit and vegetable and fat intake.[30-35] Providing automated feedback about energy balance and strategies for healthy eating have been tested for weight loss.[36,37] A randomized Internet-based weight loss trial assessed the impact of human email counseling, computer-automated tailored counseling, and no counseling and found that the human and computer-automated counseling were equally effective for weight loss at 3 months.[37] Another randomized weight loss trial assessed a self-monitoring intervention using a hand-held device with and without automated feedback and found that the subjects who

received automated feedback were significantly more likely to have lost 5% or more of body weight compared to the control group.[36]

E. Worksite social relationships have potential for widespread promotion of healthy eating behaviors. To the extent that individuals' food choices are connected, their health is also connected.[38] Understanding how food choices are associated with social relationships may help explain variation in population-level health status.[39,40] Small-scale studies have demonstrated associations between family-based food choices and friend-based food choices.[41-44] However, one large-scale study, conducted by co-I Dr. Pachucki, examined food choices among a large population of socially-tied individuals using data from the Framingham Heart Study over a ten-year period.[45] The peer diets most predictive of future food choices were "alcohol and snacks" and "healthy" patterns. Although social network analyses have demonstrated that unhealthy behaviors spread in a network,[39,40] less is known about the spread of healthy behaviors in a socially connected network.

F. Summary- Impact: A large worksite, with its established social networks, methods of communication, and shared environments, is an ideal setting for implementing strategies to prevent weight gain and reduce the rising prevalence of obesity among a large population. The project uses the worksite food environment as a platform for a weight gain prevention program that delivers personalized, automated feedback to employees through worksite communication channels over one year. Weight, health, and dietary outcomes will be assessed over two years to determine long-term effectiveness. If successful, this intervention could be applied broadly, at a relatively low cost, in other worksite, institutional, and retail (i.e. supermarket) settings to promote healthy eating and prevent obesity.

H. Innovation

1. The project intervention uses behavioral economics strategies to promote healthy food choices among a large employee population. Traditional economics assumes that individuals make rational decisions when given the appropriate information and resources to do so.[76] Most food policies and healthy eating programs are based on this premise.[77] Behavioral economics utilizes concepts from psychology to identify "decision biases" that can help explain why individuals make unhealthy choices that lead to poor health outcomes.[76] Patterns of behavior that contribute to unhealthy food choices include: doing what is customary (status quo) or going with the default option; placing disproportionate weight on the present and not considering future costs ("present-biased preferences"); being motivated by actions with immediate benefit and less motivated to achieve a long-term goal; and being influenced by what others are doing.[76,78,79] In our previous work in the MGH cafeteria, the traffic-light labeling and choice architecture intervention took advantage of some of these decision biases, including default (status quo) bias, and provided simple and salient communication about healthy food choices that did not rely on complicated, numeric nutrition information.[26-29] The project will build on this food environment and use new strategies (social norm feedback, financial incentives, and personalized feedback) to address other decision biases that commonly influence food choices, including present-biased preferences and social influence. This novel approach has not previously been tested on a large scale and is a promising strategy to change eating behaviors and prevent weight gain in a large population.

a. Social norm (peer comparison) feedback and financial incentives can increase healthy food choices. Social norm strategies have been used successfully to encourage energy conservation.[80,81] One example that is currently in practice is the "Home Energy Report" letters mailed to customers of utility companies. These letters compare a household's energy use to that of similar neighbors and provide energy conservation tips. A natural field experiment of 600,000 treatment and control household demonstrated that this program reduced energy consumption by 2.0%.[81] Evidence from small experimental studies suggests that providing informational eating norms will influence choice and quantity of food eaten.[69] Financial incentives have been shown to be effective for motivating change for multiple health behaviors,

including drug abstinence, smoking cessation, and short-term weight loss.[82-84] Price changes are the only type of financial incentives that have been tested for motivating healthy food choices, i.e. decreasing cost of healthy foods, and there is good evidence that price changes will modify the purchase of targeted foods.[85] However, it is unknown whether price changes or other types of financial incentives for healthy food purchases can influence overall dietary intake.[85]

Our research team recently completed a pilot study evaluating the effectiveness of an intervention to provide social norm feedback with and without financial incentives to increase healthy food choices among 2,700 employees at MGH who used the cafeteria regularly**.**[86] We found that the relatively "light touch" intervention of mailing letters with peer comparisons about healthy food choices and offering a small financial incentive ($10 cafeteria credit) resulted in a statistically significant increase in healthy food purchases over 3 months. These results suggest that social norm feedback with a small financial incentive program will increase healthy food choices, even among a population not actively engaged in a healthy eating program. The project will provide social norm feedback and financial incentives monthly over the course of one year. We expect that social norms in the current context will have a stronger effect than was observed in our pilot research because subjects will have actively signed up for a two-year study focusing on nutrition. To increase the potential effect of the monthly incentive, we plan to increase it from $10 to $20.

b. The intervention will provide personalized, automated feedback linking actual worksite food purchases to individual energy balance goals. Although our pilot data demonstrated that monthly letters with social norms and financial incentives could impact employees' food choices at work, a stronger approach will be needed to improve overall dietary intake at work and outside of work. In addition to monthly letters, the study intervention will provide weekly personalized emails that provide feedback about cafeteria purchases and energy balance, including daily calorie goals and physical activity calorie equivalents. Prior research on dietary feedback has relied on subjects' self-reported intake,[30-35] but our worksite food environment provides the opportunity to provide personalized feedback about calorie intake based on the employee's actual food purchases and daily caloric goals in real time. Employees will receive the feedback emails while at work and will be able to act on the information immediately in the worksite environment by making alterations in worksite food purchases and physical activity and/or adjusting their calorie consumption and activities outside of work.

2. The worksite food environment can serve as a platform to deliver long-term, scalable health promotion programs for obesity prevention. Worksite programs for obesity prevention are typically delivered over a specific period of time (e.g., 2 months) and require that the employee spend time away from their job.[15,16] Although these programs can be effective for changing employees lifestyle behaviors and weight, the long-term effectiveness and sustainability of these programs is unknown.[15,16] The project tests a new paradigm for delivering a weight gain prevention program by integrating the intervention into the daily routine of the worksite, thus lowering the time and financial burden on the employee and the employer. This type of intervention could be permanently integrated into the work environment and culture.

3. Cafeteria sales data from a large population of employees provides a unique opportunity to determine if healthy eating behaviors spread among co-workers. In the project, we will track the time and type of all food purchases of over 7,000 MGH employees who use a cafeteria debit card. From these data, we will map a network of co-workers who eat together (see Preliminary Studies below). Using these innovative methods, we can determine whether healthy eating patterns spread among co-workers and whether an intervention to promote healthy eating can influence the eating habits of co-workers socially connected to study subjects. A potential implication of this research would be to demonstrate that the positive

effects of a health promotion intervention will disseminate through socially connected co-workers, and future programs could be designed to take advantage of this phenomenon.

4. The project uses an integrated model of Social Action Theory. The project is conceptualized based on an integrated model of Social Action Theory in which the individual employee's motivation and beliefs about healthy eating are addressed in the context of the work environment and the employee's skills for making healthy food choices.[87,88] The primary outcome is change in weight at one year, and secondary outcomes are cardiovascular risk factors and dietary behaviors of employees enrolled in the study and of their socially-connected co-workers. **Figure 1** demonstrates the application of the theoretical model to the study intervention and outcomes.

Figure 1. Application of Social Action Theory to study intervention and outcomes.

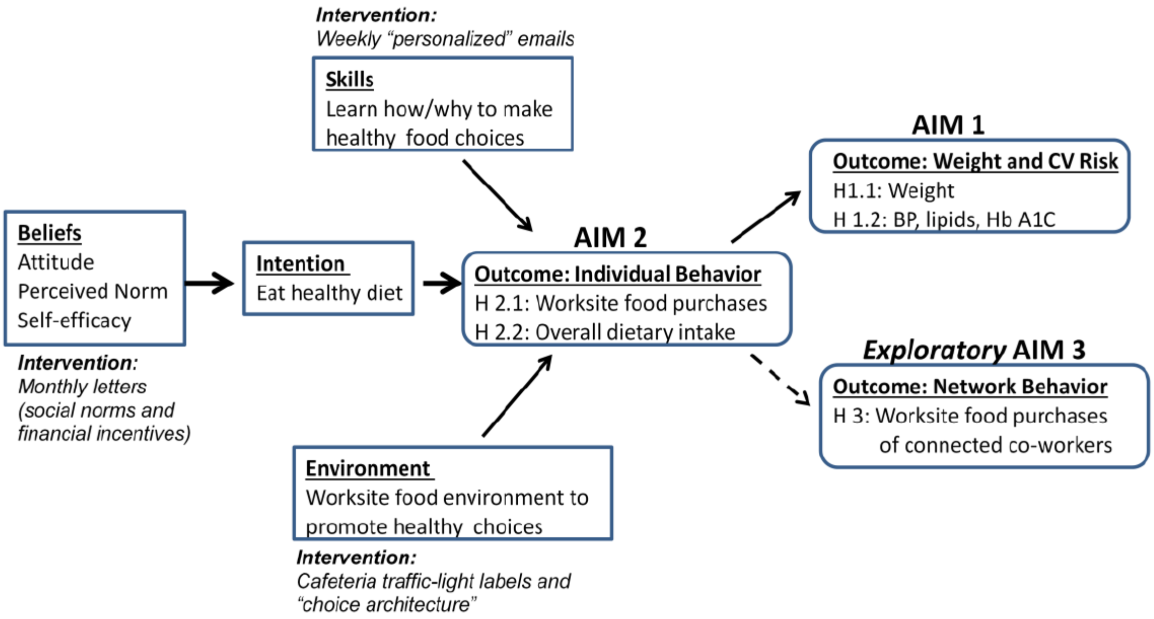

I. Preliminary studies:

1. **MGH Be Fit Program:** The MGH "Be Fit" program is a 10-week, team-based exercise and nutrition program available to employees. As part of her K23 research project, Dr. Thorndike conducted an initial evaluation of 774 employees who participated in the program and demonstrated a mean weight loss of 4.2 pounds at the end of the program and a mean weight loss of 1.0 pounds at one year follow-up.[89] This study was followed by a randomized trial of 330 employees who completed Be Fit to test a 9-month, Internet-based maintenance program.[90] Mean weight loss at one year follow up was 3.0 pounds. Dr. Thorndike has gained experience in recruiting and retaining large numbers of employees in these research studies.

2. Traffic light labeling and choice architecture cafeteria intervention: Dr. Thorndike and co-I Dr. Levy have worked together for four years to conduct research in the MGH cafeteria.[26-29] Dr. Levy was responsible for data management and analyses of cafeteria purchases. In this large cafeteria with over 6,000 daily transactions, we analyzed the effectiveness of labeling all foods and beverages as red (unhealthy), yellow (less healthy), or green (healthy) followed by a choice architecture intervention to make healthy items more convenient and visible. Results of this research showed that purchases of healthy items increased and unhealthy items decreased with traffic light labels alone, and by adding choice architecture, healthy purchases further increased.[26] These effects were consistent among all cafeteria customers, and improvements in

employees' healthy purchases were similar across all racial groups and job types.[27] Most importantly, our evaluation of the intervention at two year follow-up showed that improvements in healthy choices were sustained over time, including among a cohort of employees who visited the cafeteria regularly.[29]

3. Pilot study of social norm feedback and financial incentives: Dr. Thorndike and Dr. Levy developed a system to provide monthly social norm feedback about cafeteria purchase and financial incentives for healthy purchases in a 3-arm randomized trial. Employees were randomly assigned to one of three arms: 1) social norm feedback about healthy food purchases; 2) social norm feedback plus a small financial incentive ($10 cafeteria credit/month) to increase healthy food purchases; or 3) a control group (no contact). Employees were recruited for this study with an "opt out" strategy; therefore participants did not actively sign up to participate in a healthy eating program. Data used for social norm feedback and for study outcomes were obtained by tracking all subjects' cafeteria purchases made with their cafeteria debit card. Both of the intervention arms received a letter modeled on the "Home Energy Report" that was mailed to their home monthly for three months. Data on cafeteria purchases by all study subjects were collected for seven months (1 month baseline purchases, 3 months of intervention, 3 months of follow-up or "wash-out"). **Figure 2** shows the percentage change in the proportion of monthly "green-labeled" (healthy) purchases among the three study groups from baseline to the end of the wash-out period. Although the absolute percentage change was small, the increase in healthy purchases was significant at the end of intervention for the feedback + incentive arm (p=0.03) and borderline significant for the feedback-only arm (p=0.07) compared to the control group. The groups were not significantly different at the end of the wash-out period. During the 3-month intervention period, the 898 employees who were eligible for financial incentives earned a mean of $8.57 of cafeteria rewards per employee.

Figure 2. Change in employees' healthy food purchases during and after intervention to provide social norm feedback with and without financial incentives.

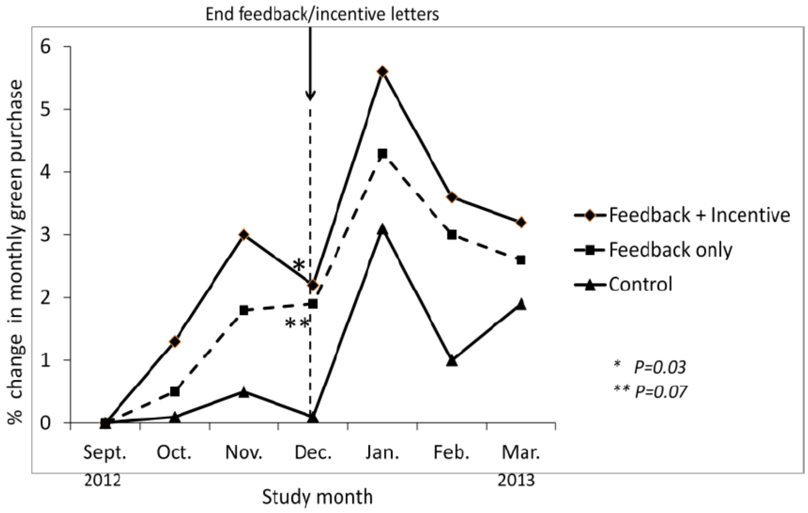

4. Traffic light labels and financial incentives to discourage sugar-sweetened beverages in a low-income, Latino grocery store: Dr. Thorndike and co-I Dr. Rimm are co-PI's for a randomized trial to discourage purchase of sugar-sweetened beverages by low-income, Latino families. They partnered with a grocery store owner to label all beverages in the store with traffic-lights and have enrolled approximately 200 families who are regular customers. Families were randomized to an intervention providing financial incentives for not purchasing sugar-sweetened beverages or to a control arm. In this project, both nutrition education and behavioral economics strategies are being utilized to change purchasing behavior, and all food purchases are being tracked with a customer loyalty card. Data collection will be completed in November 2014.

Summary of rationale for the study: The research team has extensive experience conducting studies to evaluate employee wellness programs and test behavioral economics strategies for promoting healthy food choices. Dr. Thorndike's experience with the Be Fit program demonstrates the feasibility of enrolling a large number of employees in a randomized trial, and results of Be Fit demonstrate that employees lose weight and make lifestyle changes but have difficulty in maintaining those changes over time. Given the high cost of staff-intensive, short-term wellness programs, it is clear that less expensive strategies are needed to help employees maintain a healthy weight over time. Our food environment interventions in the MGH cafeteria have demonstrated changes in healthy food choices by employees at work. The project will take advantage of the established worksite food environment, testing an intervention that does not require employees or the employer to invest a lot of time or resources. Social norm feedback and small financial incentives will increase employees' motivation to make healthier choices, and personalized feedback will increase employees' knowledge and skills to make healthier choices both at work and outside of work.

II. Specific Aims

The project is a randomized, controlled trial of 600 MGH employees to test the effectiveness of an intervention that uses the worksite food environment as a platform to deliver personalized feedback to a population of employees. The three components of the one-year intervention are: 1) automated nutrition and energy balance feedback based on an employee's food purchases and calorie goals; 2) social norm (peer comparison) feedback about worksite food purchases; and 3) financial incentives for healthy worksite food choices.

Aim 1: Determine if employees randomly assigned to the intervention group have less weight gain and lower cardiovascular risk factors than the control group at the end of intervention (1 year) and 2 year follow-up.
Hypothesis 1.1: Employees in the intervention group will maintain or lose weight and employees in the control group will gain weight at 1 year and 2-year follow up.
Hypothesis 1.2: Employees in the intervention group will have lower cholesterol, blood pressure, and hemoglobin A1c than the control group at 1 year and 2-year follow-up.
Aim 2: Determine if employees randomly assigned to the intervention group make healthier food choices than the control group at 1 year and at 2 year follow-up.
Hypothesis 2.1: Employees in the intervention group will purchase a higher proportion of healthy cafeteria items than employees in the control group.
Hypothesis 2.2: Employees in the intervention group will increase their Healthy Eating Index scores more than employees in the control group at 6 months, 1 year, and 2 year follow-up.

III. Subject Selection

A. Setting

1. Massachusetts General Hospital (MGH) is a 907-bed teaching hospital in Boston, Massachusetts with over 24,000 employees. The hospital has seven different food service locations (4 full-service cafeterias, 1 "grab and go" shop, and 2 coffee shops) on the main campus. All food service establishments will hereafter be referred to as "cafeterias" in this protocol. All MGH cafeterias are owned and operated by the hospital, and no outside food vendors are present on campus. Employees have the option of paying for all purchases with a cafeteria debit card ("platinum plate"). Over the past couple of years, the cafeteria has been transitioning from using the Platinum Plate cards to using the employee hospital ID cards for cafeteria purchases. It is anticipated that most study participants will be using the hospital ID card for cafeteria purchases by the time study enrollment begins. Purchases made with this card are directly deducted from the employee's pay check. Approximately 7,300 employees use a platinum plate card.

2. MGH cafeteria traffic light labels and choice architecture: The main cafeteria has had the traffic-light labeling system in place since 2010, and by the end of 2014, traffic-light labels will be implemented in the other six cafeterias. The traffic-light system was designed based on the USDA dietary guidelines,[92] and every item in the main cafeteria is labeled as red, yellow, or green. For the labeling system, all food and beverages are categorized into 4 groups (food entrée, food item, food condiment, or beverage) and are rated on three positive and two negative criteria. Positive criteria are: 1) fruit or vegetable as main component, 2) whole grain as main component, and 3) lean protein or low fat dairy as main component. Negative criteria are: 1) saturated fat content of ≥5 gm per entrée or ≥2 gm per item, condiment, or beverage and 2) caloric content of ≥ 500 kcal per entrée, ≥200 kcal per item, or ≥100 kcal per condiment or beverage. For beverages, each additional 100 kcal is considered an additional negative criterion. Food and beverages are categorized with the following algorithm: 1) green: positive criteria outweigh negative criteria; 2) yellow: positive criteria equal to negative criteria or possessing only one negative criterion; and 3) red: two negative criteria and no positive criteria. Items with no positive or negative criteria are rated as yellow, except for diet beverages with zero calories, which are rated green. Red beverages included sugar-sweetened beverages with ≥200 calories per container and whole milk dairy products. Yellow beverages included sugar-sweetened beverages with < 200 calories per container. Sodium content is not factored into the traffic-light system. However, sodium is listed directly on all packaged food items, and for each non-packaged item, sodium content is listed in nutrition brochures available in the cafeteria. Choice architecture changes were also implemented in the main cafeteria in 2010 to make the green-labeled foods and beverages more easily visible and convenient for purchase. These changes included re-arranging the beverage refrigerators to place the healthy beverages at eye level, placing baskets of bottled water at every food station, and re-arranging the chip racks and pre-made sandwiches to have the healthiest choices at eye level. These changes remain in place in the main cafeteria and will be implemented in the other 6 hospital cafeterias in late 2014. The food in all MGH cafeterias is prepared and labeled in a central kitchen. Although caloric content is not listed on menu boards, the traffic-light system takes calories into account, as described above. Pre-made foods that are packaged (i.e. pre-made sandwiches) do have calories and other nutritional information listed on the package. Calories for all cafeteria items are available in brochures available in the cafeterias, and this information will be utilized for the personalized feedback about calories purchased in the study intervention arm.

B. Study Subjects: All subjects will be MGH employees. Overall, employees have a mean age of 42 years, are 69% female, and are 70% white, 11% black/African American, 8% Latino, 10% Asian and < 1% Native American or Pacific Islander. The 7,300 employees who use platinum plate or ID cards to pay for cafeteria purchases have demographic characteristics similar to the overall hospital workforce.

1. Eligibility criteria:

a. Randomized trial subjects*:* Adult MGH employees (21 years or older) who use any of the MGH cafes and cafeterias four or more times a week, are willing to receive email messages through their Partners email, and pay for all cafeteria food and beverage purchases with the Platinum Plate or ID card will be eligible to enroll in the trial. For those employees who do not regularly access their Partners email through their normal work routine, study staff will provide assistance in setting up a channel for easy access such as through an encrypted smartphone, laptop, or personal desktop computer. Employees who are actively trying to gain weight, know that they will be leaving their employment at MGH within the next 12 months or will be absent from MGH for more than a month in the next year (i.e. retirement, end of training, relocation), who are currently pregnant or planning a pregnancy within the next 12 months, who have had weight loss surgery within the past 12 months or are planning a weight loss surgery during the next 12 months, who have ever had or been diagnosed with an eating disorder, or who are currently enrolled in the Be Fit 10-week employee wellness program will not be eligible to enroll

in this study. Employees who enroll in the study will not be eligible to participate in Be Fit during the two year study. In addition, cashiers at the cafes and cafeterias involved in this study (Eat Street, Coffee Central, Blossom, Tea Leaves, Riverside, Coffee South, 125 Nashua Street) will not be eligible to enroll as their job requires them to ring up purchases, which will serve as data in our study.

b. Employee network: For Aim 3, all MGH employees who use a platinum plate card for cafeteria purchases during the 1-year intervention period will be included in analyses for Aim 3.

2. Recruitment: In this project, several recruitment strategies will be utilized to enroll a diverse population of employees at MGH. These strategies are based on previous experience recruiting for the Be Fit studies and for the Platinum Plate cafeteria pilot study. The first strategy will be to send an email to all employees who use their Platinum Plate card four or more times a week, randomly sampled in waves of 25-50 employees, to introduce the study and to ask them to contact the study coordinator if they are interested in participating. From our cafeteria data, we have identified 3,000 employees who make at least 4 purchases a week at one of the hospital cafeterias, and the mean number of weekly transactions is 8.7. Following this email, follow-up letters will be sent to these employees who do not respond to the email within 2 weeks.

IV. Subject Enrollment

A. Methods of enrollment: If an employee is interested in participating in the study, study coordinators will determine eligibility by either meeting them in person or talking over the phone. In addition, study coordinators will organize information sessions for employees interested in participating. During this session, the PI and coordinator will describe the study procedures and hand out a copy of the informed consent form to each interested employee. The employee will have the opportunity to read the consent form, ask questions during the group session or individually after the session, and sign the consent form at that time or at another time to be arranged with the study staff. The consent form cover sheet will have information to contact study staff so that employees who are undecided can contact study staff at a later date to discuss enrollment and review the informed consent at a time that is convenient for the employee. To recruit employees from minority backgrounds and from some of the lower educated job types (service workers, food service workers, maintenance), the study coordinator will contact department supervisors to set up study information sessions in the department. In previous experience with the MGH Be Fit program, this has been a successful strategy to increase participation by these employee groups which have a higher proportion of non-white employees.

B. Procedures for obtaining informed consent: The study coordinator and the PI will be responsible for obtaining informed consent. Informed consent will explain that subjects will be randomized to either an intervention or a control group, and therefore there will be a 50% chance of receiving the intervention. Subjects will be informed that participation in the study will take place over a 2 year period. Subjects will be told that if they are assigned to the intervention they will receive a monthly letter at their home and weekly emails and that information collected from their cafeteria food purchases and from their baseline surveys and measurements will be used to create personalized messages. Subjects will be informed that if assigned to the intervention they will be eligible to earn small incentives to increase their healthy cafeteria purchases, but if they are assigned to control, they will not be eligible for the incentives. The consent form will outline the schedule of assessments and surveys for all study participants that take place at baseline, 6 months, 1 year, and 2 year follow-up. Subject remuneration for completing outcomes assessments will be described in detail, and these include $100 to be paid to each subject for completing each assessment at baseline, 1 year, and 2 years (survey, physical assessments, fasting blood, and two dietary recalls) and $25 for completing two 24-hour dietary recalls at 6 months. The consent form will describe that the subjects in both the

control and the intervention groups will receive 10% off all purchases made with the cafeteria Platinum Plate or ID card and that all their cafeteria purchase data will be collected by the research team. The consent form will explain that in order to receive the 10% discount in the cafeterias, subjects will need to have a study sticker placed on the back of their hospital ID or on the Platinum Plate card that will be shown to the cashier at the time that they are paying for their purchases. This discount is only to be used for cafeteria purchases made for subjects' own consumption. Periodically study staff will review purchasing history and if it appears that subjects are using this discount to pay for their co-workers' cafeteria purchases on a regular basis, study staff will contact the subject with the possibility of discontinuing the 10% discount.

As per the Partners IRB protocol, subjects and the person obtaining consent will sign two copies of the informed consent. One copy will remain on file with the Principal Investigator (PI), and the other copy will be given to the study participant. Information about contacting the study PI will be included on the consent form, and it will be explained that the subject can withdraw from the study at any time.

C. Treatment assignment and randomization: After providing informed consent and completing the baseline survey and CRC visit, the subject will be assigned to one of the two treatment conditions (intervention group or control group), stratified by weight-loss vs. weight-maintenance goals, using a computer-generated randomization scheme created by the study statistician. Study coordinators and subjects will be blinded to treatment assignment until the baseline assessment is completed.

V. Study Procedures:

After enrollment, all subjects will complete a baseline survey and two dietary recalls, in addition to undergoing a baseline physical assessment to measure weight, height, waist and hip circumference, blood pressure, resting energy expenditure, as well as a 10 mL blood draw for a fasting lipid panel, glucose, and hemoglobin A1c (HbA1c). The nurses completing the physical assessment will also conduct the IPAQ physical activity survey with participants. Over the course of the study, a total of 30 mL of blood will be drawn, 10 mL at each of the three study visits. Should subjects opt into the optional study, the total blood drawn will be 40 mL. In line with the recent NIH Accumulating Data to Optimally Predict Obesity Treatment (ADOPT) project biological recommendations (*Obesity* 2018;26:S25-S34), we will test baseline and two-year follow-up blood samples for leptin, adiponectin, and TNF-alpha. We will only perform the tests on the stored baseline samples from participants who agreed to the optional ancillary study for genotyping. At two-years, we will perform these tests plus insulin on all participants. We will not need to collect any additional blood to perform these tests. We are requesting to waive informed consent for adding these tests for the following reasons: 1) The research involves no more than minimal risk to the subjects; 2) The waiver or alteration will not adversely affect the rights and welfare of the subjects; and 3) The research could not practicably be carried out without the waiver. Since the results of these tests do not have clinically meaningful implications on an individual level, subjects will not be provided with individual results.

A. Intervention arm group

1. Monthly letters with social norm feedback and financial incentives: Subjects assigned to the intervention group will receive letters mailed to their home monthly for 12 months. These letters will report the proportion of red, yellow, and green items that the subject purchased in the cafeterias in the prior month and compare his or her purchases to "all MGH eaters" and to the "healthiest MGH eaters" (top 10% of healthy purchasers). These letters will be similar in appearance and content to the letters mailed to participants in the pilot study. The monthly letter will also include a "green goal" for the employee to achieve in the following month in order to earn a $20 financial incentive. Similar to the methods from our pilot study, financial incentives will be earned for passing each threshold proportion of 40%, 60%, or 80% green purchases in a month. For example, the October 1st letter will include a "green goal" for the

month of October. The November 1st letter will include a $20 reward if the October goal was achieved and a new green goal for the month of November. The incentive structure is designed so that employees with the least healthy purchasing patterns at baseline can earn the highest amount of money during the study. Employees who purchase green items at or above the highest threshold (80%) at the beginning of the study can earn $5 per month for remaining at that level. Therefore, employees who consistently purchase healthy items at baseline will receive a small incentive for maintaining their healthy eating patterns but will not have the opportunity to earn the $20 incentive. The maximum amount that a consistent "healthy eater" can earn during the study is $60 ($5 per month). An employee who starts below the lowest threshold of 40% green can earn a maximum of $115 over the 1 year study period ($20 x 3, achieved for passing each threshold in a single month, and $5 x 11 months for remaining at or above the final threshold). If an employee who earned the $20 reward in one month does not reach his or her goal in the next month, he or she will earn $5 for staying at or above the new threshold, but employees earn no rewards when they regress. The earned incentive money will be delivered to the employee as a check.

2. Weekly emails with personalized feedback: Two emails will be sent each week for 52 weeks. The first email of the week will include information about the study subject's prior week's cafeteria purchases, and the second email of the week will include two personalized healthy messages or "tips" that will focus on healthy eating, physical activity, and disease prevention. The subject lines for these emails will not contain any personal information. The first email will be called "Your ChooseWell 365 weekly report," and the second will be called "Your ChooseWell 365 weekly tips." The email messages will include five personalized components:

i) <u>Daily calorie goals</u>: At baseline, all subjects will complete a resting energy expenditure measurement to estimate daily calorie goals, and subjects in the intervention group will opt for either a weight-maintenance or a weight-loss calorie goal that will be included in the email messages. Weight loss goals will be estimated to be 500 calories lower than the weight maintenance goals, but no subject will be given a goal of less than 1200 calories per day.

ii) <u>Cafeteria purchases:</u> For each day of the week, all cafeteria purchases will be listed with total calories purchased for each day compared to the daily caloric goal and highlighting calories "available" for consumption outside of work. The caloric content and red, yellow, or green label for individual food items purchased will be listed for each day of the week. Providing detailed information about calories purchased in the context of daily caloric goals will provide a benchmark to guide their food choices not only at work but also outside of work.

iii) <u>Physical activity</u>: These messages will focus on the role of physical activity in maintaining the daily calorie goal. Previous research has suggested that providing physical activity equivalents of calorie contents in foods can increase healthy food and beverage choices.[93,94] Messages about physical activity will vary each week and will be tailored to the individual by providing 1) calorie equivalents for activities that can be done at work (i.e. walking up 10 flights of stairs in the Yawkey Building; walking around the perimeter of the MGH campus) and 2) physical activity equivalents (in walking distances) for calorie content of unhealthy (red-labeled) foods they had purchased that week. All intervention subjects will be provided with a link to an on-campus walking map with distances.

iv) <u>Barriers to healthy eating and activity</u>: Weekly emails will include personalized messages (based on weight, calorie goals, job type, and cafeteria purchasing patterns) that target barriers to healthy eating and physical activity, such as portion size, lack of time, skipping meals, lack of sleep, and a sedentary job.

v) <u>Medical and family history</u>: Weekly emails will include personalized messages related to medical and family history obtained from the baseline survey. Specific messages will target diagnoses of hypertension, hyperlipidemia, diabetes, cardiovascular disease, and a family history of cardiovascular disease.

vi) <u>Contact information for study staff:</u> Every email will provide an email and phone number that participants can contact with questions about messaging or requests to change daily caloric goals and weight goals (which will be reflected in subsequent personalized messaging). Subjects will be able to change their goal from weight loss to maintenance, or vice-versa, at any time during the intervention.

During Year 1 of the project, the study nutritionist, Emily Gelsomin, RD, will work with Dr. Thorndike and Dr. Eric Rimm to develop the content of messages. The computer programmer and Dr. Levy will create the logic that will use the static baseline data and the changing cafeteria purchase data to develop a series of emails that would be directly relevant to the study subject.

As part of the messaging intervention development, during Year 1 we will conduct a brief pilot study on salad bar usage at Eat Street, the main cafeteria at the hospital, in order to determine the average number of calories per ounce of salad purchased. While 13.6% of purchases by frequent cafeteria users include a salad from the salad bar, the cash register data describing food purchases do not provide any information on the contents of these salads besides their weight. Given the great variety of items available at the salad bar, from leafy greens and beans to cheeses and dressings, it is impossible to estimate an average caloric content of a salad purchase without doing this pilot work. Study nutritionist, Emily Gelsomin, and study coordinator, Nathan Weil, will survey 130 MGH employees who purchase a salad at the Eat Street Cafe. Study staff will approach the employee after paying for their salad at the cash register. This is a similar approach that our team utilized to survey cafeteria customers in 2010. Only customers wearing MGH employee ID badges will be approached. Study staff will inform employees of the purpose of the pilot study and ask if they would like to participate. Study staff will additionally inform customers that all questions are optional, participation will only take under three minutes, and, at the end of the survey, they will be given a coupon for a free coffee at any MGH cafe for their participation. The study staff will ask the employee the survey questions, including gender, age category, race/ethnicity, and hospital department where they are employed. We will not record any personally identifiable information such as name or employee ID number. Each survey will be assigned a record number used only for this pilot study. The study staff will then record all contents in the salad use a pre-designed survey rubric, marking each item and approximate quantity on a list of all available salad bar items. Once each item in the salad has been accounted for study staff will record the weight of the salad using a portable scale. Once the survey is complete, the employee will be given a coupon for a free MGH coffee. To analyze these data we will run a multiple regression with calories as dependent variable, weight in ounces as independent variable, and demographic indicators as covariates.

B. Control arm group

Subjects assigned to the control group will receive monthly letters during the 1 year intervention period that include general nutrition information. All control subjects will receive the same letter each month.

C. Study follow-up for subjects who leave MGH

Participants who leave their employment at MGH prior to the end of the one-year intervention period and follow-up visit are discontinued from the study because they are not able to participate in the worksite intervention. However, participants who complete the one-year intervention period and visit but who leave their employment at MGH during the following year will be given the option of completing the two-year follow-up visit, online survey, and online 24-hour dietary recalls if they are able to come back to MGH and willing to complete the surveys. Study coordinators will contact these participants using a cell phone number provided at the consent meeting. Since participants who left MGH no longer have Partners' email addresses, study coordinators will request permission to use their personal email addresses to send secured links to the REDCap survey and 24-hour dietary recalls. While participant cell phone numbers will be the primary means of contact for scheduling the follow-up visits, study

coordinators will use the participants' personal email addresses for appointment invitations and reminders, as well as for resolving any scheduling issues.

VI. Biostatistical Analysis

A. Aim 1: Determine if employees assigned to the intervention group have less weight gain and lower cardiovascular risk factors than the control group at the end of intervention (1 year) and 2-year follow up.

1. Hypothesis 1.1: Employees in the intervention group will maintain or lose weight and employees in the control group will gain weight at 1 year and 2-year follow-up.

a. Data collection and measures: A survey and physical assessment will be completed by all subjects at baseline, 1 year (end of intervention), and 2 year follow-up.

<u>Survey:</u> All subjects will complete a baseline survey electronically (or a paper version if the subject does not have computer access) to provide subject's and subject's immediate family's medical history, subject's medications, smoking history, physical activity level, eating habits and behaviors, perceived stress, use of the Internet, demographic background, and body weight goal for the year (i.e. maintain current weight, lose weight). The same survey will be repeated at one year and 2 years with static questions (i.e. demographics) removed in the follow-up surveys.

<u>Physical assessments and fasting blood:</u> Assessments will be performed by research nurses in the Clinical Research Center (CRC) at MGH. The CRC is centrally located in the main hospital building and will be convenient for most employees. If an employee is unable to visit the CRC for an assessment due to job responsibilities or unforeseen circumstances, a research nurse will be able to meet them at a convenient location to complete the assessment. The employee will be asked to fast for 12 hours prior to the assessment. Assessments will include measurement of the subject's weight, height, waist and hip circumference, and blood pressure. Physical activity will be measured with the International Physical Activity Questionnaire.[95] Blood measurements will include a lipid panel (total cholesterol, LDL, HDL, and triglycerides), glucose, and HbA1c. Resting energy expenditure will be measured using the VMAX Encore 29 metabolic cart, Viasys Healthcare, Carefusion, San Diego, CA, using best practice methods.[96] All measurements will be repeated at 1-year and 2-year follow-up, with the exception of the resting energy expenditure assessment which will only be performed at the baseline visit.

b. Outcomes: Primary outcomes will be change in weight at 1 year and 2 years follow-up compared to baseline. Secondary outcomes will be change in waist circumference and body mass index.

c. Analysis: We will compare the change in weight between intervention and control groups at the end of 1-year and 2-year follow-up using two-sample t-tests. We will also use repeated measures analyses to combine data from both time points using mixed effects models. A time-treatment interaction term will be included in the models. We will evaluate 1) the overall average treatment effect across 1 year and 2 years and 2) the treatment group difference in the change from baseline to 1 year and from baseline to 2 years. The known predictors of weight change will also be included in the mixed effects models to improve the precision of parameter estimates.

d. Power calculation: A total of 670 subjects will be recruited. From a previous study,[90] we expect a <10% attrition rate and we do not expect the attrition rate to be different between the two study groups since change of employment was the main reason for loss-to-follow-up in our prior study. The final sample size for analysis will be 540, or approximately 270 per group. Using the data from our prior worksite exercise and nutrition studies, we estimate the standard deviation in weight change at 1 year is about 5 pounds. With 270 subjects per arm, the study will have 93% power to detect a mean difference as small as 1.5 lbs between the two study arms with a two-sided significance level of 0.05.

2. Hypothesis 1.2: Employees in the intervention group will have lower cholesterol, blood pressure, and hemoglobin A1c than the control group at 1 year and 2-year follow-up.

a. Outcomes: Outcomes will be change in total cholesterol, blood pressure, and HbA1c at 1 year and 2 years follow-up compared to baseline. Secondary outcomes will be change in waist circumference, LDL cholesterol, triglycerides, and glucose at 1 year and 2 years compared to baseline.

b. Analysis: We will compare the changes in the primary and secondary outcomes between intervention and control groups at the end of 1-year and 2-year follow-up using two-sample t-tests or Wilcoxon rank sum tests, whichever more appropriate. We will also use repeated measures analyses to combine data from both time points using mixed effects models. A time-treatment interaction term will be included in the models. We will evaluate 1) the overall average treatment effect across 1 year and 2 years and 2) the treatment group difference in the change from baseline to 1 year and from baseline to 2 years. The known predictors of outcomes will also be included in the mixed effects models to improve the precision of parameter estimates.

c. Power calculation: Estimates for standard deviations are based on 1-year results in our prior worksite studies.[60,61] Assuming standard deviation for total cholesterol change in 1 year is 30 mg/dL, the study will have 90% power to detect a mean difference of 8.4 mg/dL. Assuming standard deviation for blood pressure change in 1 year is 10 mmHg, the study will have 90% power to detect a mean difference of 2.8 mmHg. Assuming standard deviation for HbA1C change in 1 year is 1, the study will have 90% power to detect a mean difference of 0.28.

B. Aim 2: Determine if employees assigned to the intervention group make healthier food choices at follow up than the control group.

1. Hypothesis 2.1: Employees in the intervention group purchase a higher proportion of healthy cafeteria items than employees in the control group.

a. Data collection: Data will be collected from cash register sales data. Beginning in November 2009, the cash registers in the main cafeteria were programmed to collect specific names of all cafeteria items so they could be identified as green, yellow, or red in the research database. For platinum plate users, purchases can be linked to employee's identification number, demographics, department, and job type. In 2014, the cash registers in the other 6 food service establishments on the MGH campus were programmed to collect purchasing data. When the intervention starts, purchasing data from all cafeteria sales will be downloaded to the study database on a weekly basis throughout the 2-year study period. Baseline data will be obtained by analyzing cafeteria purchases over the three months prior to the of the one year intervention period.

b. Measures: We will assess the proportion of red, yellow, and green-labeled items purchased across all MGH cafeterias for each subject in each month of the study from baseline (one month prior to initiating the intervention) through the 12 months of intervention and during the 12 months of post-intervention follow-up.

c. Outcomes: Primary outcomes will be changes in the proportions of green- and red-labeled items purchased by a subject in the baseline month compared to month 12 (end of intervention). Secondary outcomes will be changes from baseline to month 24 (end of follow-up), as well as changes in the proportions of green- and red-labeled beverages from baseline to months 12 and 24.

d. Analysis: We will use two-sample t-tests to compare the mean change in the monthly proportion of green (or red) items purchased from baseline to month 12 for the intervention versus control groups. Linear regression models will be used to control for employee-specific variables that, despite randomization, are out of balance between the two groups. We will similarly assess changes from baseline to month 24 to determine whether the intervention

effects are sustained over time. We will extend these models to explore whether race or education modifies the effect of the interventions by testing appropriate interaction terms.

e. Power calculation: Our pilot study of social norms and financial incentives demonstrated approximately 3% increase in green purchases after 3 months.[86] Based on these results, assuming the standard deviation for change in proportion of green purchases is 20%, the study will have 82% power to detect a mean difference of 5 percentage points between the intervention group and the control group at 1 year with a two-sided significance level of 0.05.

2. Hypothesis 2.2: Employees in the intervention group increase their Healthy Eating Index score more than employees in the control group at 6 months, 1 year, and 2 year follow-up.

a. Data collection: Each subject will complete two separate Automated Self-Administered 24-hour dietary recalls (ASA24) several days apart at 4 time points: baseline, 6 months, 1 year, and 2 year follow up. The ASA24 is a freely-available web-based software tool that is modeled on an interviewer-administered method developed by the United States Department of Agriculture that uses multi-level food probes to accurately assess food types and amounts.[97] The advantages of using the ASA24 are that it can be administered to a large population at a low cost and that it is a valid measure of overall mean intake of nutrients and summary diet measures in an adult population.[98,99]

b. Measures: The Health Eating Index (HEI) is a tool to measure compliance with the key, diet-related recommendations of the United States Department of Agriculture's Dietary Guidelines for Americans.[100,101] It was first developed in 1995 and has been revised in 2005 (HEI-5) and 2010 (HEI-10) to comply with updates in the US Dietary Guidelines. The HEI measures dietary intake based on density rather than quantity of foods consumed. Total scores can range from 0-100, with 100 being the healthiest diet. Higher HEI scores are associated with lower risk of chronic disease, including cardiovascular disease, diabetes, and cancer in both women and men.[102,103] National data from 2007-2008 showed a mean overall HEI-5 for Americans of 53.5, a number similar to what was found in 2001-2002.[104] An HEI-10 score can be improved by increasing intake of fruits and vegetables, low fat or fat-free milk, whole grains, and seafood and by reducing foods high in solid fats and added sugars.[101] The traffic-light labeling system in the MGH cafeteria was developed based on 2005 Dietary Guidelines and remains consistent with the updated 2010 guidelines. To estimate HEI scores, the two 24-hour dietary recalls from each assessment period will be combined using methodology described by Freedman, et al.,[105] and will have less bias than those estimated from a single assessment.

c. Outcomes: Primary outcomes will be change in the total HEI-10 score from baseline to 12 months (end of intervention). Secondary outcomes will be change from baseline to 6 months and to month 24 (end of follow-up), as well as change in HEI-10 component scores for empty calories, fatty acids, refined grains, whole grains, total fruit, and total vegetables from baseline to months 6, 12, and 24.

d. Analysis: We will estimate the HEI score from the two ASA24 dietary recalls collected at baseline, 6 months, 1 year, and 2 years based on methodology described in the Freedman article.[64] Mean change in total HEI-10 score from baseline to 1 year and from baseline to 2 years will be compared using similar methodology as described above for changes in proportion of green foods. Secondary analyses will compare mean change in sub-categories of the HEI-10 score from baseline to 6 months and mean change in HEI-10 component scores (e.g. Total Fruit, Total Vegetables, Whole Grains, Sodium, and Empty Calories). We will also use repeated measures analyses to combine data from all three time points to determine whether there is an overall difference between the two study arms, or whether there is a difference in trend over time.

e. Power calculation: Chiuve et al. (with co-I Dr. Rimm) demonstrated a reduced risk of chronic disease with increasing quintiles of HEI scores, and an increase of 5 points in HEI score was sufficient to move a subject into a higher quintile.[102] Therefore, we consider a 5-point

difference to be a meaningful dietary change. Assuming the standard deviation for change in HEI score is 15, the study will have 97% power to detect a mean difference of 5 between the two study arms with 270 subjects in each arm.

Analysis Plan: Updated June 17, 2019
Data will be analyzed using SAS version 9.4.

Primary analyses will be performed on an intent-to-treat basis that includes all randomized participants. Missing data could occur in several scenarios: (1) Loss to follow-up due to leaving employment, which is expected to have occurred at random in both groups and to be unrelated to the study outcomes; (2) Failure to attend a follow-up appointment or a visit to obtain a weight measurement; and (3) Censoring due to pregnancy. For participants who became pregnant during the intervention year, their follow-up values on all outcomes will be considered missing. We will impute missing data using multiple imputation and employing all available outcomes assessments and all prior data including baseline data, HEI conducted measured at 6 months, and all purchasing data prior to 2-year follow-up. We will conduct two sensitivity analyses: (1) including only participants who completed follow-up assessment at 1 year and (2) including only participants who remained employed at the hospital during the one-year intervention. If sensitivity analyses yield substantively different results, we will conduct exploratory analyses to assess reasons for the difference.

Hypothesis 1.1: The primary outcome is change in weight (kg) at the end of one year. Change in weight at the end of two years is a secondary outcome. Other secondary outcomes include *percent* body weight lost and gained less than 1 lb. We will compare the change in weight between intervention and control groups using a difference-in-differences approach. To improve the precision of the effect estimate, we will use a linear/logistic regression analysis including age, sex, race, ethnicity, education, job type, and physical activity in the model.

We will use a repeated measures approach to combine data from 1-year and 2-year weight outcomes. A mixed effect model with a random effect for subject and a time treatment interaction will be used to examine the intervention effect at each time point and over the entire period.

Hypothesis 1.2: We will compare the changes in each of the cardiometabolic risk factors (waist circumference, systolic and diastolic blood pressure, fasting glucose, HbA1c, triglycerides, LDL and HDL cholesterol, total cholesterol) between intervention and control groups at the end of 1-year and 2-year follow-up using a difference-in-differences approach. The analysis strategy will be identical to the one used for the primary outcome in Hypothesis 1.1.

Hypothesis 2.1: We will compare the healthfulness of food and beverages purchased at work between the intervention and control groups. Outcomes will include the average proportion of items purchased that are labeled green and labeled red, the average Healthy Purchasing Score, and the average number of calories of purchased foods during each one-year period (intervention period and follow-up period) compared to the baseline period (one year prior to enrollment). The analysis strategy will be identical to the one used for the primary outcome in Hypothesis 1.1.

Secondary analyses will explore the trend of these outcomes. We will explore whether the intervention effects diminish over time using a repeated measures approach. The mixed effect models will include a random effect for subject and a time treatment interaction fixed effect to determine whether the intervention effects differ among quarters or whether any trend exists.

Hypothesis 2.2: We will compare the change in dietary intake (via the Healthy Eating Index) of the intervention and control groups during the intervention year using 3 follow-up time points: 6 months, 1 year, and 2 years. We will utilize a mixed effects model that includes a random effect for subject and a time treatment interaction fixed effect to examine the intervention effect at the two time points separately. We will also add age, sex, race, ethnicity, education, and job type to the model to increase the precision of effect estimate.

VII. Risks and Discomforts

For all subjects, including the larger population of employees included in Aim 3 of the project, procedures to protect the privacy and to maintain confidentiality of data include the following: 1) Access to subject information will be protected by a Partners secure server. External access to such information is blocked by secure external firewalls. 2) Subjects will be assigned a unique study identification number. All data and the key linking the study identification number to the subject's information will be maintained on a Partners secure server that is password protected. 3) Identifiable study data will not be analyzed or published in any fashion that provides the ability to identify subjects.

Dr. Thorndike will screen all blood test results and will contact subjects individually to inform them of any critical results and to refer them for appropriate medical follow-up.

The risks of the study procedures are minimal. Study participants will be monitored for the occurrence of events defined as any undesirable experience. All adverse events will be reported in accordance with the Partners Healthcare policies and guidelines. These reports will include a description of all undesirable experiences, required interventions, a participant's condition after an event, an estimate of the extent of injury, and potential strategies to prevent future occurrences. The PI will classify the relationship of the study protocol to the event and will be responsible for reporting serious adverse events (death, life threatening, new, serious or permanent disability) to the Institutional Review Board. Unanticipated problems involving risks to subjects or others including adverse events will be reported to the Partners IRB within 5 working days/7 calendar days of the date the investigator first becomes aware of the problem, which is in accordance with Partners IRB guidelines for reporting unanticipated problems, including adverse events. Serious adverse events will be reported to the NIH in accordance with stated policies of reporting fatal or life-threatening unexpected, suspected serious adverse reactions within 7 days and non life-threatening unexpected, suspected serious adverse reactions within 15 days of the receipt of information.

VIII. Potential Benefits

Study subjects may not receive any direct benefit from being in the research study. It is possible that subjects will improve their dietary intake and their clinical risk factors, such as lowering cholesterol or blood pressure and preventing weight gain. It is possible that employees who are socially-connected to employees participating in the trial will improve their eating habits at work. The results of this research could provide a new model for other employers and institutions to deliver long-term health promotion interventions to prevent obesity among large populations of workers. In the future, results of this research could lead to new strategies to promote healthier food choices by utilizing food purchasing data to provide nutrition feedback, and these strategies could be used in retail settings.

IX. Monitoring and Quality Assurance

The risks of the study are minimal, and therefore there is no formal Data Safety Monitoring Board planned. Dr. Thorndike will be available to all study subjects for questions or concerns regarding the study. Dr. Thorndike will review all blood test results to screen for abnormalities and "critical" results. She will review all safety data with Dr. Levy and the

statistician, Dr. Yuchiao Chang, on a monthly basis. Standard protocols for reporting serious adverse events to the Partners IRB and the NIH will be followed. All adverse events and subject complaints will be reported to the IRB in the annual continuing review. Any breach of confidentiality or privacy would be considered an adverse event and will be reported to the Partners IRB.

X. References

1. Finucane MM. Stevens GA. Cowan MJ. Danaei G. Lin JK. Paciorek CJ. Singh GM. Gutierrez HR. Lu Y. Bahalim AN. Farzadfar F. Riley LM. Ezzati M. Global Burden of Metabolic Risk Factors of Chronic Diseases Collaborating Group (Body Mass Index). National, regional, and global trends in body-mass index since 1980: Systematic analysis of health examination surveys and epidemiological studies with 960 country-years and 9.1 million participants. *Lancet*. 2011;377(9765):557-567.

2. Wang YC, McPherson K, Marsh T, Gortmaker SL, Brown M. Health and economic burden of the projected obesity trends in the USA and the UK. *Lancet*. 2011;378(9793):815-825. https://phstwlp1.partners.org:2443/login?url=http://ovidsp.ovid.com/ovidweb.cgi?T=JS&CSC=Y&NEWS=N&PAGE=fulltext&D=medl&AN=21872750.

3. McCrory MA, Suen VM, Roberts SB. Biobehavioral influences on energy intake and adult weight gain. *J Nutr*. 2002;132(12):3834S.

4. Gortmaker SL, Swinburn BA, Levy D, et al. Changing the future of obesity: Science, policy, and action. *Lancet*. 2011;378(9793):838-847.

5. Lewis CE, Jacobs DR,Jr, McCreath H, et al. Weight gain continues in the 1990s: 10-year trends in weight and overweight from the CARDIA study. coronary artery risk development in young adults. *Am J Epidemiol*. 2000;151(12):1172-1181.

6. Swinburn B, Egger G. Preventive strategies against weight gain and obesity. *Obes Rev*. 2002;3(4):289-301.

7. Swinburn BA, Sacks G, Hall KD, et al. The global obesity pandemic: Shaped by global drivers and local environments. *Lancet*. 2011;378(9793):804-814.

8. Hill JO, Wyatt HR, Reed GW, Peters JC. Obesity and the environment: Where do we go from here? *Science*. 2003;299(5608):853-855. doi: 10.1126/science.1079857 [doi].

9. Bennett GG, Foley P, Levine E, et al. Behavioral treatment for weight gain prevention among black women in primary care practice: A randomized clinical trial. *JAMA Intern Med*. 2013;173(19):1770-1777. doi: 10.1001/jamainternmed.2013.9263 [doi].

10. Lemon SC, Wang ML, Wedick NM, et al. Weight gain prevention in the school worksite setting: Results of a multi-level cluster randomized trial. *Prev Med*. 2014;60:41-47. doi: 10.1016/j.ypmed.2013.12.010 [doi].

11. Wolfenden L, Wyse R, Nichols M, Allender S, Millar L, McElduff P. A systematic review and meta-analysis of whole of community interventions to prevent excessive population weight gain. *Prev Med*. 2014;62:193-200. doi: 10.1016/j.ypmed.2014.01.031 [doi].

12. Beauchamp A, Backholer K, Magliano D, Peeters A. The effect of obesity prevention interventions according to socioeconomic position: A systematic review. *Obes Rev*. 2014;15(7):541-554. doi: 10.1111/obr.12161 [doi].

13. Anderko L, Roffenbender JS, Goetzel RZ, et al. Promoting prevention through the affordable care act: Workplace wellness. *Prev Chronic Dis*. 2012;9:E175.

14. Carnethon M. Whitsel LP. Franklin BA. Kris-Etherton P. Milani R. Pratt CA. Wagner GR. American Heart Association Advocacy Coordinating Committee. Council on Epidemiology and Prevention. Council on the Kidney in Cardiovascular Disease. Council on Nutrition, Physical Activity and Metabolism. Worksite wellness programs for cardiovascular disease prevention: A policy statement from the american heart association. *Circulation*. 2009;120(17):1725-1741.

15. Anderson LM, Quinn TA, Glanz K, et al. The effectiveness of worksite nutrition and physical activity interventions for controlling employee overweight and obesity: A systematic review. *Am J Prev Med*. 2009;37(4):340-357.

16. Engbers LH, van Poppel MN, Chin A Paw, M J, van Mechelen W. Worksite health promotion programs with environmental changes: A systematic review. *Am J Prev Med*. 2005;29(1):61-70.

17. Pelletier KR. Clinical and cost outcomes of multifactorial, cardiovascular risk management interventions in worksites: A comprehensive review and analysis. *J Occup Environ Med*. 1997;39(12):1154-1169.

18. Pelletier KR. A review and analysis of the clinical and cost-effectiveness studies of comprehensive health promotion and disease management programs at the worksite: Update VII 2004-2008. *J Occup Environ Med*. 2009;51(7):822-837.

19. Thorndike AN. Workplace interventions to reduce obesity and cardiometabolic risk. *Curr Cardiovasc Risk Rep*. 2011;5:79-85.

20. Baicker K, Cutler D, Song Z. Workplace wellness programs can generate savings. *Health affairs*. 2010;29(2):1-8.

21. Caloyeras J, Liu H, Exum E, Broderick M, Mattke S. Managing maifest diseases, but not health risks, saved PepsiCo money over seven years. *Health Aff (Millwood)*. 2014;33:124-131.

22. Engbers LH, van Poppel MN, Chin A Paw M, van Mechelen W. The effects of a controlled worksite environmental intervention on determinants of dietary behavior and self-reported fruit, vegetable and fat intake. *BMC Public Health*. 2006;6:253.

23. Raulio S, Roos E, Prattala R. School and workplace meals promote healthy food habits. *Public Health Nutr*. 2010;13(6A):987-992.

24. Lowe MR, Tappe KA, Butryn ML, et al. An intervention study targeting energy and nutrient intake in worksite cafeterias. *Eat behav*. 2010;11(3):144-151.

25. Thornton LE, Lamb,K.E., Ball,K. Employment status, residential and workplace food environments: Associations with women's eating behaviors. *Health and Place*. 2013;24:80-89.

26. Thorndike AN, Sonnenberg L, Riis J, Barraclough S, Levy DE. A 2-phase labeling and choice architecture intervention to improve healthy food and beverage choices. *Am J Public Health*. 2012;102(3):527-533.

27. Levy DE, Riis J, Sonnenberg LM, Barraclough SJ, Thorndike AN. Food choices of minority and low-income employees: A cafeteria intervention. *Am J Prev Med*. 2012;43(3):240-248.

28. Sonnenberg L, Gelsomin E, Levy DE, Riis J, Barraclough S, Thorndike AN. A traffic light food labeling intervention increases consumer awareness of health and healthy choices at the point-of-purchase. *Preventive medicine*. 2013;57:253-257.

29. Thorndike AN, Riis J, Sonnenberg LM, Levy DE. Traffic-light labels and choice architecture: Promoting healthy food choices. *Am J Prev Med*. 2014;46:143-149.

30. Brug J, Steenhuis I, van Assema P, de Vries H. The impact of a computer-tailored nutrition intervention. *Prev Med*. 1996;25(3):236-242.

31. Kroeze W, Werkman A, Brug J. A systematic review of randomized trials on the effectiveness of computer-tailored education on physical activity and dietary behaviors. *Ann Behav Med*. 2006;31(3):205-223.

32. Papadaki A, Scott JA. Follow-up of a web-based tailored intervention promoting the mediterranean diet in scotland. *Patient Educ Couns*. 2008;73(2):256-263.

33. Atienza AA, King AC, Oliveira BM, Ahn DK, Gardner CD. Using hand-held computer technologies to improve dietary intake. *Am J Prev Med*. 2008;34(6):514-518.

34. Wright JL, Sherriff JL, Dhaliwal SS, Mamo JC. Tailored, iterative, printed dietary feedback is as effective as group education in improving dietary behaviours: Results from a randomised control trial in middle-aged adults with cardiovascular risk factors. *Int j behav nutr phys act*. 2011;8:43.

35. Broekhuizen K, Kroeze W, van Poppel MN, Oenema A, Brug J. A systematic review of randomized controlled trials on the effectiveness of computer-tailored physical activity and dietary behavior promotion programs: An update. *Ann Behav Med*. 2012;44(2):259-286.

36. Burke LE, Conroy MB, Sereika SM, et al. The effect of electronic self-monitoring on weight loss and dietary intake: A randomized behavioral weight loss trial. *Obesity (Silver Spring)*. 2011;19(2):338-344. doi: 10.1038/oby.2010.208 [doi].

37. Tate DF, Jackvony EH, Wing RR. A randomized trial comparing human e-mail counseling, computer-automated tailored counseling, and no counseling in an internet weight loss program. *Arch Intern Med*. 2006;166(15):1620-1625. doi: 166/15/1620 [pii].

38. Smith KP, Christakis NA. Social networks and health. *Annu Rev Sociol*. 2008;34:405-429.

39. Christakis NA, Fowler JH. The spread of obesity in a large social network over 32 years. *N Engl J Med*. 2007;357(4):370-379.

40. Christakis NA, Fowler JH. The collective dynamics of smoking in a large social network. *N Engl J Med*. 2008;358(21):2249-2258.

41. Bove CF, Sobal J, Rauschenbach BS. Food choices among newly married couples: Convergence, conflict, individualism, and projects. *Appetite*. 2003;40(1):25-41.

42. Sobal J, Nelson MK. Commensal eating patterns: A community study. *Appetite*. 2003;41(2):181-190.

43. Rozin P, Riklis J, Margolis L. Mutual exposure or close peer relationships do not seem to foster increased similarity in food, music or television program preferences. *Appetite*. 2004;42(1):41-48.

44. Young ME, Mizzau M, Mai NT, Sirisegaram A, Wilson M. Food for thought. what you eat depends on your sex and eating companions. *Appetite*. 2009;53(2):268-271.

45. Pachucki MA, Jacques PF, Christakis NA. Social network concordance in food choice among spouses, friends, and siblings. *Am J Public Health*. 2011;101(11):2170-2177.

46. Guerrieri R, Nederkoorn C, Stankiewicz K, et al. The influence of trait and induced state impulsivity on food intake in normal-weight healthy women. *Appetite*. 2007;49(1):66-73. doi: S0195-6663(06)00665-9 [pii].

47. Guerrieri R, Nederkoorn C, Jansen A. The effect of impulsive personality on overeating and obesity: Current state of affairs. *Psychological Topics*. 2008;17:265-286.

48. Guerrieri R, Nederkoorn C, Jansen A. How impulsiveness and variety influence food intake in a sample of healthy women. *Appetite*. 2007;48(1):119-122. doi: S0195-6663(06)00509-5 [pii].

49. Salmon SJ, Fennis BM, de Ridder DT, Adriaanse MA, de Vet E. Health on impulse: When low self-control promotes healthy food choices. *Health Psychol*. 2014;33(2):103-109. doi: 10.1037/a0031785 [doi].

50. Nederkoorn C, Guerrieri R, Havermans RC, Roefs A, Jansen A. The interactive effect of hunger and impulsivity on food intake and purchase in a virtual supermarket. *Int J Obes (Lond)*. 2009;33(8):905-912. doi: 10.1038/ijo.2009.98 [doi].

51. Hofmann W, Friese M, Strack F. Impulse and self-control from a dual-systems perspective. *Perspect Psychol Sci*. 2009;4(2):162-176. doi: 10.1111/j.1745-6924.2009.01116.x [doi].

52. Michie S, Abraham C, Whittington C, McAteer J, Gupta S. Effective techniques in healthy eating and physical activity interventions: A meta-regression. *Health Psychol*. 2009;28(6):690-701. doi: 10.1037/a0016136 [doi].

53. Rising CJ, Bol N. Nudging our way to a healthier population: The effect of calorie labeling and self-control on menu choices of emerging adults. *Health Commun*. 2016:1-7. doi: 10.1080/10410236.2016.1217452 [doi].

54. Will Crescioni A, Ehrlinger J, Alquist JL, et al. High trait self-control predicts positive health behaviors and success in weight loss. *J Health Psychol*. 2011;16(5):750-759. doi: 10.1177/1359105310390247 [doi].

55. Lawyer SR, Boomhower SR, Rasmussen EB. Differential associations between obesity and behavioral measures of impulsivity. *Appetite*. 2015;95:375-382. doi: 10.1016/j.appet.2015.07.031 [doi].

56. van der Laan, L N, Barendse ME, Viergever MA, Smeets PA. Subtypes of trait impulsivity differentially correlate with neural responses to food choices. *Behav Brain Res*. 2016;296:442-450. doi: 10.1016/j.bbr.2015.09.026 [doi].

57. VanderBroek-Stice L, Stojek MK, Beach SR, vanDellen MR, MacKillop J. Multidimensional assessment of impulsivity in relation to obesity and food addiction. *Appetite*. 2017;112:59-68. doi: S0195-6663(16)30754-1 [pii].

58. Solanto MV, Abikoff H, Sonuga-Barke E, et al. The ecological validity of delay aversion and response inhibition as measures of impulsivity in AD/HD: A supplement to the NIMH multimodal treatment study of AD/HD. *J Abnorm Child Psychol*. 2001;29(3):215-228.

59. Epstein LH, Salvy SJ, Carr KA, Dearing KK, Bickel WK. Food reinforcement, delay discounting and obesity. *Physiol Behav*. 2010;100(5):438-445. doi: 10.1016/j.physbeh.2010.04.029 [doi].

60. Tangney JP, Baumeister RF, Boone AL. High self-control predicts good adjustment, less pathology, better grades, and interpersonal success. *J Pers*. 2004;72(2):271-324. doi: 263 [pii].

61. Roberto CA, Bragg MA, Schwartz MB, et al. Facts up front versus traffic light food labels: A randomized controlled trial. *Am J Prev Med*. 2012;43(2):134-141.

62. Veling H, Aarts H, Papies EK. Using stop signals to inhibit chronic dieters' responses toward palatable foods. *Behav Res Ther*. 2011;49(11):771-780. doi: 10.1016/j.brat.2011.08.005 [doi].

63. Veling H, van Koningsbruggen GM, Aarts H, Stroebe W. Targeting impulsive processes of eating behavior via the internet. effects on body weight. *Appetite*. 2014;78:102-109. doi: 10.1016/j.appet.2014.03.014 [doi].

64. Enax L, Hu Y, Trautner P, Weber B. Nutrition labels influence value computation of food products in the ventromedial prefrontal cortex. *Obesity (Silver Spring)*. 2015;23(4):786-792. doi: 10.1002/oby.21027 [doi].

65. Hare TA, Camerer CF, Rangel A. Self-control in decision-making involves modulation of the vmPFC valuation system. *Science*. 2009;324(5927):646-648. doi: 10.1126/science.1168450 [doi].

66. Hare TA, Malmaud J, Rangel A. Focusing attention on the health aspects of foods changes value signals in vmPFC and improves dietary choice. *J Neurosci*. 2011;31(30):11077-11087. doi: 10.1523/JNEUROSCI.6383-10.2011 [doi].

67. Leary MR, Kelly KM, Cottrell CA, Schreindorfer LS. Construct validity of the need to belong scale: Mapping the nomological network. *J Pers Assess*. 2013;95(6):610-624. doi: 10.1080/00223891.2013.819511 [doi].

68. Robinson E, Otten R, Hermans RCJ. Descriptive peer norms, self-control and dietary behavior in young adults. *Psychology and Health*. 2016;31:9-20.

69. Robinson E, Thomas J, Aveyard,P., Higgs,S. What everyone else is eating: A systematic review and meta-analysis of the effect of informational eating norms on eating behavior. *J Acad Nutr Diet*. 2013.

70. U.S. Department of Health and Human Services, Office of Disease Prevention and Health Promotion. National action plan to improve health literacy [internet]. . 2013:Available from: http://health.gov/communication/HLActionPlan/.

71. Lipkus IM, Samsa G, Rimer BK. General performance on a numeracy scale among highly educated samples. *Med Decis Making*. 2001;21(1):37-44.

72. Nelson W, Reyna VF, Fagerlin A, Lipkus I, Peters E. Clinical implications of numeracy: Theory and practice. *Ann Behav Med*. 2008;35(3):261-274. doi: 10.1007/s12160-008-9037-8 [doi].

73. Schwartz LM, Woloshin S, Black WC, Welch HG. The role of numeracy in understanding the benefit of screening mammography. *Ann Intern Med*. 1997;127(11):966-972.

74. Rothman RL, Housam R, Weiss H, et al. Patient understanding of food labels: The role of literacy and numeracy. *Am J Prev Med*. 2006;31(5):391-398.

75. VanEpps EM, Downs JS, Loewenstein G. Calorie label formats: Using numeric and traffic light calorie labels to reduce lunch calories. *Journal of Public Policy and Marketing*. 2016;35:26-36.

76. Loewenstein G, Brennan T, Volpp KG. Asymmetric paternalism to improve health behaviors. *JAMA*. 2007;298(20):2415-2417.

77. Krukowski RA, Harvey-Berino J, Kolodinsky J, Narsana RT, Desisto TP. Consumers may not use or understand calorie labeling in restaurants. *J Am Diet Assoc*. 2006;106(6):917-920.

78. Thaler RH, Sunstein CR. *Nudge: Improving decisions about health, wealth, and happiness.* New York, New York: Penguin Group; 2009.

79. Miles S, Scaife V. Optimistic bias and food. *Nutrition Research Reviews*. 2003;16:3-19.

80. Goldstein NJ, Cialdini RB, Griskevicius V. A room with a viewpoint: Using social norms to motivate environmental conservation in hotels. *Journal of Consumer Research*. 2008;35.

81. Allcott H. Social norms and energy conservation. *Journal of Public Economics*. 2011;95:1082-1095.

82. Higgins ST, Wong CJ, Badger GJ, Ogden DEH, Dantona RL. Contingent reinforcement increases cocaine abstinence during outpatient treatment and 1 year follow-up. *J of Consulting and Clinical Psychology*. 2000;68:64-72.

83. Volpp KG, John LK, Troxel AB, Norton L, Fassbender J, Loewenstein G. Financial incentive-based approaches for weight loss: A randomized trial. *JAMA*. 2008;300(22):2631-2637.

84. Volpp KG, Troxel AB, Pauly MV, et al. A randomized, controlled trial of financial incentives for smoking cessation. *N Engl J Med*. 2009;360(7):699-709.

85. Epstein LH, Jankowiak N, Nederkoorn C, Raynor HA, French SA, Finkelstein E. Experimental research on the relation between food price changes and food-purchasing patterns: A targeted review. *Am J Clin Nutr*. 2012;95(4):789-809.

86. Thorndike AN, Riis J, Levy DE. Peer comparison feedback and financial incentives to promote employees' healthy food choices: A randomized controlled trial. *Circulation*. 2014;129:[moderated poster presentation, American Heart Association Epi/NPAM Scientific Sessions, March 2014].

87. Ewart CK. Social action theory for a public health psychology. *Am Psychol*. 1991;46(9):931-946.

88. Fishbein M, Yzer MC. Using theory to design effective health behavior interventions. *Communication Theory*. 2003;13(2):164-183.

89. Thorndike AN, Healey E, Sonnenberg L, Regan S. Participation and cardiovascular risk reduction in a voluntary worksite nutrition and physical activity program. *Preventive medicine*. 2011;52:164-166.

90. Thorndike AN, Sonnenberg L, Healey E, Myint-U K, Kvedar JC, Regan S. Prevention of weight gain following a worksite nutrition and exercise program: A randomized controlled trial. *Am J Prev Med*. 2012;43(1):27-33. https://phstwlp1.partners.org:2443/login?url=http://ovidsp.ovid.com/ovidweb.cgi?T=JS&CSC=Y&NEWS=N&PAGE=fulltext&D=medl&AN=22704742.

91. Matthews LJ, DeWan P, Rula EY. Methods for inferring health-related social networks among coworkers from online communication patterns. *PLoS ONE*. 2013;8(2):e55234.

92. United States Department of Agriculture. Dietary guidelines for americans. http://www.mypyramid.gov/guidelines/index.html. Updated 2005. Accessed June 11, 2009.

93. Bleich SN, Herring BJ, Flagg DD, Gary-Webb TL. Reduction in purchases of sugar-sweetened beverages among low-income black adolescents after exposure to caloric information. *Am J Public Health*. 2012;102(2):329-335. doi: 10.2105/AJPH.2011.300350 [doi].

94. Dowray S, Swartz JJ, Braxton D, Viera AJ. Potential effect of physical activity based menu labels on the calorie content of selected fast food meals. *Appetite*. 2013;62:173-181. doi: 10.1016/j.appet.2012.11.013 [doi].

95. Craig CL, Marshall AL, Sjostrom M, et al. International physical activity questionnaire: 12-country reliability and validity. *Med Sci Sports Exerc*. 2003;35(8):1381-1395.

96. Compher C, Frankenfield D, Keim N, Roth-Yousey L, Evidence Analysis Working Group. Best practice methods to apply to measurement of resting metabolic rate in adults: A systematic review. *J Am Diet Assoc*. 2006;106(6):881-903. doi: S0002-8223(06)00146-5 [pii].

97. National Cancer Institute. ASA24 automated self-administered 24 hour recall. http://riskfactor.cancer.gov/tools/instruments/asa24/. Accessed Feb. 1, 2014.

98. Frankenfeld CL, Poudrier JK, Waters NM, Gillevet PM, Xu Y. Dietary intake measured from a self-administered, online 24-hour recall system compared with 4-day diet records in an adult US population. *J Acad Nutr Diet*. 2012;112(10):1642-1647.

99. Subar AF, Kirkpatrick SI, Mittl B, et al. The automated self-administered 24-hour dietary recall (ASA24): A resource for researchers, clinicians, and educators from the national cancer institute. *J Acad Nutr Diet*. 2012;112(8):1134-1137.

100. Guenther PM, Reedy J, Krebs-Smith SM. Development of the healthy eating index-2005. *J Am Diet Assoc*. 2008;108(11):1896-1901.

101. Guenther PM, Casavale KO, Reedy J, et al. Update of the healthy eating index: HEI-2010. *J Acad Nutr Diet*. 2013;113(4):569-580.

102. Chiuve SE, Fung TT, Rimm EB, et al. Alternative dietary indices both strongly predict risk of chronic disease. *J Nutr*. 2012;142(6):1009-1018.

103. George SM, Ballard-Barbash R, Manson JE, et al. Comparing indices of diet quality with chronic disease mortality risk in postmenopausal women in the women's health initiative observational study: Evidence to inform national dietary guidance. *Am J Epidemiol*. 2014. doi: kwu173 [pii].

104. Guenther PM, Casavale KO, Kirkpatrick SI, et al, eds. *Diet quality of americans in 2001-02 and 2007-08 as measured by the healthy eating index-2010.* Alexandria, VA: United States Department of Agriculture Center for Nutrition Policy and Promotion; 2013Nutrition Insight 51.

105. Freedman LS, Guenther PM, Krebs-Smith SM, Dodd KW, Midthune D. A population's distribution of healthy eating index-2005 component scores can be estimated when more than one 24-hour recall is available. *J Nutr*. 2010;140(8):1529-1534.

106. Marsden PV. Core discussion networks of americans. *American Sociological Review*. 1987;52:122-131.

107. O'Malley AJ. The analysis of social network data: An exciting frontier for statisticians. *Stat Med*. 2013;32(4):539-555. doi: 10.1002/sim.5630 [doi].

108. Manski CF. Identification of endogenous social effects: The reflection problem. *Review of Economic Studies*. 1993;60(3):531-542.

109. Christakis NA, Fowler JH. Social contagion theory: Examining dynamic social networks and human behavior. *Stat Med*. 2013;32(4):556-577. doi: 10.1002/sim.5408 [doi].

110. Patton JH, Stanford MS, Barratt ES. Factor structure of the barratt impulsiveness scale. *J Clin Psychol*. 1995;51(6):768-774.

111. Fortenbaugh FC, DeGutis J, Germine L, et al. Sustained attention across the life span in a sample of 10,000: Dissociating ability and strategy. *Psychol Sci*. 2015;26(9):1497-1510. doi: 10.1177/0956797615594896 [doi].

112. Riley E, Okabe H, Germine L, Wilmer J, Esterman M, DeGutis J. Gender differences in sustained attentional control relate to gender inequality across countries. *PLoS One*. 2016;11(11):e0165100. doi: 10.1371/journal.pone.0165100 [doi].

113. Odum AL. Delay discounting: I'm a k, you're a k. *J Exp Anal Behav*. 2011;96(3):427-439. doi: 10.1901/jeab.2011.96-423 [doi].

114. Best JR, Theim KR, Gredysa DM, et al. Behavioral economic predictors of overweight children's weight loss. *J Consult Clin Psychol*. 2012;80(6):1086-1096. doi: 10.1037/a0029827 [doi].

115. Barlow P, Reeves A, McKee M, Galea G, Stuckler D. Unhealthy diets, obesity and time discounting: A systematic literature review and network analysis. *Obes Rev*. 2016;17(9):810-819. doi: 10.1111/obr.12431 [doi].

116. Carlson SM, Moses LJ. Individual differences in inhibitory control and children's theory of mind. *Child Dev*. 2001;72(4):1032-1053.

117. Hunt MK, Hopko DR, Bare R, Lejuez CW, Robinson EV. Construct validity of the balloon analog risk task (BART): Associations with psychopathy and impulsivity. *Assessment*. 2005;12(4):416-428. doi: 12/4/416 [pii].

118. White TL, Lejuez CW, de Wit H. Test-retest characteristics of the balloon analogue risk task (BART). *Exp Clin Psychopharmacol*. 2008;16(6):565-570. doi: 10.1037/a0014083 [doi].

119. Lejuez CW, Read JP, Kahler CW, et al. Evaluation of a behavioral measure of risk taking: The balloon analogue risk task (BART). *J Exp Psychol Appl*. 2002;8(2):75-84.

120. Bornovalova MA, Daughters SB, Hernandez GD, Richards JB, Lejuez CW. Differences in impulsivity and risk-taking propensity between primary users of crack cocaine and primary users of heroin in a residential substance-use program. *Exp Clin Psychopharmacol*. 2005;13(4):311-318. doi: 2005-15800-005 [pii].

121. Osborn CY, Weiss BD, Davis TC, et al. Measuring adult literacy in health care: Performance of the newest vital sign. *Am J Health Behav*. 2007;31 Suppl 1:36. doi: 10.5555/ajhb.2007.31.supp.S36 [doi].

122. Weiss BD, Mays MZ, Martz W, et al. Quick assessment of literacy in primary care: The newest vital sign. *Ann Fam Med*. 2005;3(6):514-522. doi: 3/6/514 [pii].
