# Supplementary material for: Automated Behavioral Workplace Intervention to Prevent Weight Gain and Improve Diet: The ChooseWell 365 Randomized Clinical Trial
Source: JAMA Netw Open. 2021 Jun 7;4(6):e2112528. doi: 10.1001/jamanetworkopen.2021.12528 (PMC8185595; doi:10.1001/jamanetworkopen.2021.12528)
Supplement: Supplement 2. — eTable 1. Examples of Personalized Messages Emailed to Intervention Group eTable 2. Number of ASA24 Dietary Recalls Completed by Participants eTable 3. Subgroup Analysis 1: Changes in Weight and Dietary Measures by Weight Loss Goals eTable 4. Subgroup Analysis 2: Changes in Weight and Dietary Measures by Baseline BMI Category eTable 5. Subgroup Analysis 3: Changes in Weight and Dietary Measures by International Physical Activity Questionnaire Score Tertile eFigure 1. Intervention Components Rated Helpful for Making Healthier Choices by Intervention Participants eFigure 2. Total Number of Intervention Components Rated Helpful by Intervention Participants Who Rated 1 or More Components [file jamanetwopen-e2112528-s002.pdf]

Supplemental Online Content

Thorndike AN, McCurley JL, Gelsomin ED, et al. Automated behavioral workplace intervention to prevent weight gain and improve diet: the ChooseWell 365 randomized clinical trial. *JAMA Netw Open*. 2021;4(6):e2112528. doi:10.1001/jamanetworkopen.2021.12528

eTable 1. Examples of Personalized Messages Emailed to Intervention Group

eTable 2. Number of ASA24 Dietary Recalls Completed by Participants

eTable 3. Subgroup Analysis 1: Changes in Weight and Dietary Measures by Weight Loss Goals

eTable 4. Subgroup Analysis 2: Changes in Weight and Dietary Measures by Baseline BMI Category

eTable 5. Subgroup Analysis 3: Changes in Weight and Dietary Measures by International Physical Activity Questionnaire Score Tertile

eFigure 1. Intervention Components Rated Helpful for Making Healthier Choices by Intervention Participants

eFigure 2. Total Number of Intervention Components Rated Helpful by Intervention Participants Who Rated 1 or More Components

This supplemental material has been provided by the authors to give readers additional information about their work.

© 2021 Thorndike AN et al. *JAMA Network Open.*

eTable 1. Examples of Personalized Messages Emailed to Intervention Group

Domain	Sub-domain	Data source	Message trigger	Personalized Message Examples
**Weight and energy balance**	Maintaining Weight	Cafeteria purchase	Purchase of unhealthy beverages	You purchased at least two red-labeled beverages last week. Red beverages typically contain more than 200 calories and regularly consuming them may put you at risk for weight gain.
	Losing Weight	Cafeteria purchase	Purchase of tomato basil mozzarella panini	Last week you purchased a tomato basil mozzarella panini. Although it does contain a serving of vegetables, it is high in calories. For weight loss, look for green panini options, or other green-labeled cafeteria entrees like the hot veggie wrap at the Chef's Stage.
	Losing Weight	Baseline Visit	IPAQ score	Getting more exercise can help you lose weight. The goal is 150 minutes a week and walking counts! Try adding a 10 or 15-minute walk to your day.
**Disease risk**	Hypertension	Baseline survey and cafeteria purchase	History of hypertension and purchase of BLT sandwich	Did you know that your recent BLT purchase exceeded the amount of sodium you should have in an entire day? For a lower sodium option, add extra vegetables to your sandwich and avoid processed meats like bacon and ham.
	High Cholesterol	Baseline survey	Taking cholesterol medication	Want to try to reduce your cholesterol medication? Look for MGH cafeterias green-labeled options: they contain whole grains, fruits, vegetables, and lean proteins--regular consumption of these foods may help lower your cholesterol.
	Family history CVD	Baseline survey and cafeteria purchase	Family history cardiovascular disease and purchase of slice of pizza	Just one slice of pizza is high in sodium and saturated fat. Over time, this may increase your risk of developing heart disease. Try a green-labeled entree at the Carvery instead; these options are typically much healthier for your heart.
**Workplace food purchases**	Coffee shop	Cafeteria purchase	Purchase of Jumbo Muffin Coffee Combo	Did you know having a jumbo muffin with your coffee (or tea) is like eating a slice of cake for breakfast? (It has a similar calorie content and about as much fat and carbohydrate as a cake slice.) For a better-balanced option, try the Be Fit muffin combo next time.
	Main cafeteria	Cafeteria purchase	Purchase at the breakfast "bar" (weighted items)	Most Americans fall short of meeting the recommendation to eat about 2 cups of fruit per day. Try to aim for a cup of fruit (about the size of your fist) in the morning. The breakfast bar offers a variety of fruit and can help you meet this goal.
	Main cafeteria	Cafeteria purchase	Purchase of whole wheat veggie pizza slice	Your recent whole wheat veggie pizza selection was a good choice. Although it has similar calories to a slice of cheese pizza, it is a good source of fiber, providing about 15% of your total fiber requirement for the day.
**Home food choices**	Takeout/ restaurant meals	Baseline survey	Eating out or getting takeout	Substitute a side salad for an order of fries when you are out to eat to increase your intake of

© 2021 Thorndike AN et al. *JAMA Network Open.*

**and behaviors**			more than once a week	vegetables and save an average of 200 calories per meal.
	Meal preparation	Baseline survey	One or more people <18 years old living in the household	Reducing the number of sodas, juices, and sports drinks at home can be good for the whole family -- like many adults, children often get too many calories from sugary beverages. (For a healthier soda alternative, try mixing fruit juice with sparkling water.)
	Meal preparation	Baseline survey	>1 person living in household and eat out/takeout > 1x per week	Do you find preparing meals for one person is a challenge? Use frozen vegetables as a quick, convenient way to balance any dinner. Aim to have vegetables fill 1/2 your plate.
**Barriers to healthy eating**	Nutrition knowledge	Baseline survey	Incorrect assessment of what green cafeteria labels mean	Did you know that the green-labeled items in MGH cafeterias are calorie-controlled and contain whole grains, lean proteins, and/or fruits and vegetables? These items represent the healthiest options offered at MGH.
	Time management	Baseline survey	Caring for disabled or ill person	You are busy taking care of others, but don't forget to take care of yourself too: if you don't have time to prepare a healthy meal, look for the green flags in our cafeteria. They help guide you towards the healthiest options at MGH.
	Boredom	Baseline survey	Shops for household groceries more than half the time	Try mixing up your meal planning by visiting sites like Cooking Light and Eating Well. You can search for fresh quick meal options and swap out any old recipes that are growing stale. Links: http://www.cookinglight.com and http://www.eatingwell.com/
**Physical activity**	Work-related	Baseline survey	Uses stairs sometimes, rarely or never when going places at work	Did you know that walking 4 flights of stairs at MGH three times per day will burn an extra 100 calories (or more) on average? This is one way to increase your activity at work.
	Work-related	Baseline survey	Purchase of chocolate cake	You recently purchased chocolate cake. You would have to walk to White 10 over eight times to burn off those calories. Try to reduce how often you purchase this item (or similar items) and increase your physical activity to help prevent weight gain over time.
	Disease prevention	Baseline survey	IPAQ score	Studies show exercising fewer than 3 times per week can increase the likelihood of developing diabetes. The good news: being more active can help reduce your risk (and walking counts).

Abbreviations: BLT, bacon, lettuce, and tomato; IPAQ, International Physical Activity Questionnaire; MGH, Massachusetts General Hospital

© 2021 Thorndike AN et al. *JAMA Network Open.*

eTable 2. Number of ASA24 Dietary Recalls Completed by Participants

Number ASA24's completed	Intervention group, (N=299)	Control group (N=303)
Baseline		
Two ASA24	262 (87.6%)	265 (87.4%)
One ASA 24	27 (9.0%)	23 (7.6%)
None: did not complete	10 (3.3%)	15 (5.0%)
6-month follow-up		
Two ASA24	194 (64.9%)	198 (65.3%)
One ASA 24	48 (16.1%)	37 (12.2%)
None: did not complete	48 (16.1%)	64 (21.1%)
None: discontinued study[a]	9 (3.0%)	4 (1.3%)
12-month follow-up		
Two ASA24	231 (77.3%)	222 (73.3%)
One ASA 24	25 (8.4%)	21 (6.9%)
None: did not complete	25 (8.4%)	36 (11.9%)
None: discontinued study[a]	18 (6.0%)	24 (7.9%)
24-month follow-up		
Two ASA24	222 (74.2%)	217 (71.6%)
One ASA 24	13 (4.3%)	14 (4.6%)
None: did not complete	29 (9.7%)	39 (12.9%)
None: discontinued study[a]	35 (11.7%)	33 (10.9%)

Abbreviation: ASA24, Automated Self-Administered 24-hour dietary recall.

[a] Reasons for discontinuing study included leaving employment, participant withdrawal, and ineligibility due to starting new job in the cafeteria.

© 2021 Thorndike AN et al. *JAMA Network Open.*

eTable 3. Subgroup Analysis 1: Changes in Weight and Dietary Measures by Weight Loss Goals

	**Goal: lose weight (N=503)**			**Goal: maintain weight (N=99)**			
	Intervention group, Mean (95% CI)	Control group, Mean (95% CI)	P-value	Intervention group, Mean (95% CI)	Control group, Mean (95% CI)	P-value	P-value, study group x weight goal
Weight, kgs							
12-month Δ	0.6 (0.0, 1.1)	0.2 (-0.4, 0.8)	0.33	0.7 (-0.3, 1.7)	1.4 (0.5, 2.2)	0.31	0.25
24-month Δ	1.4 (0.7, 2.1)	0.6 (-0.2, 1.4)	0.16	1.9 (0.4, 3.3)	1.6 (0.7, 2.5)	0.73	0.72
BMI, kg/m^2							
12-month Δ	0.2 (0.0, 0.4)	0.1 (-0.1, 0.3)	0.38	0.2 (-0.1, 0.6)	0.5 (0.2, 0.8)	0.30	0.28
24-month Δ	0.5 (0.2, 0.8)	0.3 (-0.0, 0.5)	0.20	0.6 (0.1, 1.1)	0.6 (0.2, 0.9)	0.82	0.69
Green-labeled items, %							
12-month Δ[a]	9.4 (8.1, 10.8)	2.4 (1.2, 3.6)	<0.001	8.9 (4.7, 13.2)	-0.1 (-2.9, 2.7)	<0.001	0.39
24-month Δ[b]	5.2 (3.5, 6.9)	1.2 (-0.4, 2.9)	<0.001	8.1 (4.0, 12.2)	-0.9 (-4.4, 2.6)	0.001	0.093
Red-labeled items, %							
12-month Δ[a]	-4.9 (-5.7, -4.1)	-1.1 (-1.8, -0.3)	<0.001	-4.1 (-6.4, -1.8)	-0.1 (-1.4, 1.3)	0.004	0.87
24-month Δ[b]	-3.8 (-4.7, -2.8)	-1.3 (-2.4, -0.3)	<0.001	-5.5 (-8.0, -2.9)	0.3 (-2.7, 3.3)	0.003	0.077
HPS, %							
12-month Δ[a]	7.2 (5.0, 9.4)	1.7 (0.9, 2.6)	<0.001	6.5 (3.5, 9.6)	-0.0 (-1.8, 1.8)	<0.001	0.50
24-month Δ[b]	4.5 (1.7, 7.3)	1.3 (0.1, 2.4)	<0.001	6.8 (3.7, 9.6)	-0.6 (-3.6, 2.4)	<0.001	0.051
HEI-15 Score, Mean (SD)							
12-month Δ	1.5 (-0.3, 3.4)	-1.2 (-3.1, 0.8)	0.045	-1.2 (-4.3, 1.9)	0.6 (-3.3, 4.6)	0.45	0.15
24-month Δ	0.1 (-1.9, 2.0)	-1.2 (-3.2, 0.8)	0.37	1.4 (-1.8, 4.7)	-1.2 (-5.5, 3.2)	0.33	0.71

Abbreviations: BMI, Body mass index; HPS, Healthy purchasing score; HEI-15, Healthy Eating Index-15 Score

[a] Compares purchases during 12-month intervention period (months 1 to 12) to purchases during 12-month baseline period (12 months pre-intervention).

[b] Compares purchases during 12-month follow-up period (months 13 to 24) to purchases during 12-month baseline period (12 months pre-intervention).

© 2021 Thorndike AN et al. *JAMA Network Open.*

eTable 4. Subgroup Analysis 2: Changes in Weight and Dietary Measures by Baseline BMI Category

	**BMI<25 kg/m²** (N=224)			**BMI 25-29.9 kg/m²** (N=195)			**BMI ≥ 30 kg/m²** (N=182)			
	Intervention group, Mean (95% CI)	Control group, Mean (95% CI)	P-value	Intervention group, Mean (95% CI)	Control group, Mean (95% CI)	P-value	Intervention group, Mean (95% CI)	Control group, Mean (95% CI)	P-value	P-value, study group x BMI category
Weight, kgs										
12-month Δ	0.7 (0.2, 1.1)	0.6 (0.2, 1.1)	0.77	0.6 (-0.3, 1.5)	0.6 (-0.3, 1.4)	0.96	0.5 (-0.5, 1.5)	-0.1 (-1.5, 1.2)	0.46	0.76
24-month Δ	1.6 (0,7, 2.4)	1.1 (0.6, 1.7)	0.38	2.0 (0.8, 3.1)	0.7 (-0.2, 1.6)	0.12	0.9 (-0.5, 2.4)	0.3 (-2.0, 2.5)	0.53	0.85
BMI, kg/m²										
12-month Δ	0.2 (0.1, 0.4)	0.2 (0.0, 0.4)	0.86	0.2 (-0.1, 0.5)	0.2 (-0.1, 0.5)	0.92	0.2 (-0.1, 0.5)	-0.0 (-0.5, 0.5)	0.52	0.82
24-month Δ	0.5 (0.3, 0.8)	0.4 (0.2, 0.6)	0.42	0.7 (0.3, 1.1)	0.7 (0.3, 1.1)	0.17	0.3 (0.1, 0.6)	0.1 (-0.7, 0.9)	0.57	0.88
Green-labeled items, %										
12-month Δ[a]	9.3 (6.8, 11.7)	1.3 (-0.4, 3.0)	<0.001	10.2 (8.0. 12.4)	3.2 (1.4, 5.1)	<0.001	8.7 (6.3, 11.2)	1.8 (-0.4, 4.1)	<0.001	0.78
24-month Δ[b]	5.7 (3.1, 8.4)	1.1 (-1.3, 3.4)	0.006	6.1 (2.9, 9.2)	2.8 (0.4, 5.2)	0.061	5.3 (2.7, 7.9)	-1.4 (-4.4, 1.6)	0.002	0.74
Red-labeled items, %										
12-month Δ[a]	-4.4 (-5.8, -3.1)	-0.9 (-1.8, -0.1)	<0.001	-5.3 (-6.8, -3.8)	-0.8 (-2.0, 0.5)	<0.001	-4.7 (-5.9, -3.4)	-1.3 (-2.8, 0.2)	<0.001	0.42
24-month Δ[b]	-4.0 (-5.6, -2.5)	-1.3 (-2.9, 0.3)	0.011	-3.7 (-5.6, -1.9)	-1.3 (-2.9, 0.2)	0.019	-4.4 (-5.7, -3.1)	-0.5 (-2.8, 1.8)	0.008	0.95
HPS, %										
12-month Δ[a]	6.9 (5.1, 8.6)	1.1 (0.0, 2.2)	<0.001	7.7 (6.1, 9.4)	2.0 (0.6, 3.4)	<0.001	6.7 (5.0, 8.4)	1.6 (-0.1, 3.2)	<0.001	0.72
24-month Δ[b]	4.9 (3.0, 6.8)	1.2 (-0.6, 2.9)	0.003	4.9 (2.7, 7.1)	2.1 (0.5, 3.7)	0.018	4.8 (3.2, 6.5)	-0.4 (-2.8, 1.9)	<0.001	0.81
HEI-15 Score										
12-month Δ	1.1 (-1.4, 3.5)	-1.7 (-4.4, 1.1)	0.14	-0.5 (-3.7, 2.6)	0.5 (-2.6, 3.5)	0.45	2.7 (-0.2, 5.5)	-1.6 (-5.2, 2.0)	0.044	0.11
24-month Δ	1.6 (-0.7, 3.9)	-3.8 (-6.5, -1.2)	0.002	-2.2 (-5.2, 0.8)	2.0 (-1.2, 5.2)	0.029	1.4 (-2.2, 4.9)	-1.1 (-4.9, 2.7)	0.28	0.002

Abbreviations: BMI, Body mass index; HPS, Healthy purchasing score; HEI-15, Healthy Eating Index-15 Score

[a] Compares purchases during 12-month intervention period (months 1 to 12) to purchases during 12-month baseline period (12 months pre-intervention).

[b] Compares purchases during 12-month follow-up period (months 13 to 24) to purchases during 12-month baseline period (12 months pre-intervention).

© 2021 Thorndike AN et al. *JAMA Network Open.*

eTable 5. Subgroup Analysis 3: Changes in Weight and Dietary Measures by International Physical Activity Questionnaire Score Tertile

	**IPAQ tertile 1 0-2,619 MET-minutes/week (N=200)**			**IPAQ tertile 2 2,627-6,777 MET-minutes/week (N=201)**			**IPAQ tertile 3 6,816-42,579 MET-minutes/week (N=200)**			
	Intervention, Mean (95% CI)	Control, Mean (95% CI)	P-value	Intervention, Mean (95% CI)	Control, Mean (95% CI)	P-value	Intervention, Mean (95% CI)	Control, Mean (95% CI)	P-value	P-value, inter-vention x tertile
Weight, kgs										
12-month Δ	0.4 (-0.3, 1.2)	0.3 (-0.8, 1.4)	0.84	0.4 (-0.4, 1.2)	0.5 (-0.1, 1.1)	0.90	0.9 (0.0, 1.8)	0.3 (-0.5, 1.2)	0.35	0.74
24-month Δ	0.7 (-0.1, 1.5)	0.8 (-1.0, 2.5)	0.93	1.4 (0.3, 2.5)	0.5 (-0.3, 1.3)	0.20	2.5 (1.0, 3.9)	1.0 (0.2, 1.9)	0.083	0.43
BMI, kg/m2										
12-month Δ	0.2 (-0.1, 0.4)	0.1 (-0.3, 0.5)	0.93	0.2 (-0.1, 0.4)	0.2 (-0.0, 0.4)	0.92	0.3 (0.0, 0.6)	0.1 (-0.2, 0.4)	0.36	0.74
24-month Δ	0.2 (-0.0, 0.5)	0.3 (-0.3, 0.9)	0.80	0.5 (0.1, 0.9)	0.2 (-0.1, 0.5)	0.18	0.8 (0.4, 1.3)	0.4 (0.1, 0.7)	0.12	0.40
Green-labeled items, %										
12-month Δ[a]	10.2 (8.1, 12.4)	1.8 (-0.0, 3.6)	<0.001	9.0 (6.6, 11.3)	1.3 (-0.8, 3.3)	<0.001	8.9 (6.4, 11.5)	3.0 (1.2, 4.9)	<0.001	0.48
24-month Δ[b]	7.1 (4.6, 9.6)	-0.7 (-2.9, 1.5)	<0.001	5.1 (2.4, 7.8)	0.4 (-2.6, 3.5)	0.023	5.0 (2.0, 8.0)	3.1 (0.7, 5.5)	0.33	0.089
Red-labeled items, %										
12-month Δ[a]	-4.7 (-6.1, -3.4)	-0.6 (-1.8, 0.5)	<0.001	-4.9 (-6.2, -3.6)	-0.6 (-1.9, 0.7)	<0.001	-4.7 (-6.1, -3.3)	-1.4 (-2.6, -0.3)	<0.001	0.70
24-month Δ[b]	-4.2 (-5.5, -2.8)	0.4 (-1.2, 1.9)	<0.001	-4.5 (-6.2, -2.8)	-1.4 (-3.3, 0.5)	0.015	-3.6 (-5.2, -1.9)	-2.2 (-4.0, -0.5)	0.28	0.15
HPS, %										
12-month Δ[a]	7.5 (5.9, 9.0)	1.2 (-0.1, 2.5)	<0.001	6.9 (5.3, 8.6)	0.9 (-0.6, 2.4)	<0.001	6.8 (5.0, 8.6)	2.2 (1.0, 3.5)	<0.001	0.50
24-month Δ[b]	5.7 (3.9, 7.4)	-0.5 (-2.1, 1.1)	<0.001	4.8 (2.8, 6.8)	0.9 (-1.4, 3.2)	0.010	4.3 (2.3, 6.3)	2.7 (0.9, 4.4)	0.23	0.058
HEI-15 Score										
12-month Δ[a]	1.2 (-1.7, 4.2)	-1.2 (-4.5, 2.0)	0.27	0.2 (-2.3, 2.6)	-0.5 (-3.5, 2.4)	0.71	1.8 (-1.2, 4.8)	-1.0 (-4.1, 2.1)	0.20	0.75
24-month Δ[b]	0.0 (-3.4, 3.4)	-1.4 (-4.6, 1.9)	0.56	0.0 (-2.7, 2.7)	0.3 (-3.0, 3.5)	0.91	0.9 (-1.8, 3.7)	-2.6 (-5.6, 0.5)	0.095	0.48

Abbreviations: BMI, Body mass index; HPS, Healthy purchasing score; HEI-15, Healthy Eating Index-15 Score

[a] Compares purchases during 12-month intervention period (months 1 to 12) to purchases during 12-month baseline period (12 months pre-intervention).

[b] Compares purchases during 12-month follow-up period (months 13 to 24) to purchases during 12-month baseline period (12 months pre-intervention).

© 2021 Thorndike AN et al. *JAMA Network Open.*

eFigure 1. Intervention Components Rated Helpful for Making Healthier Choices by Intervention Participants

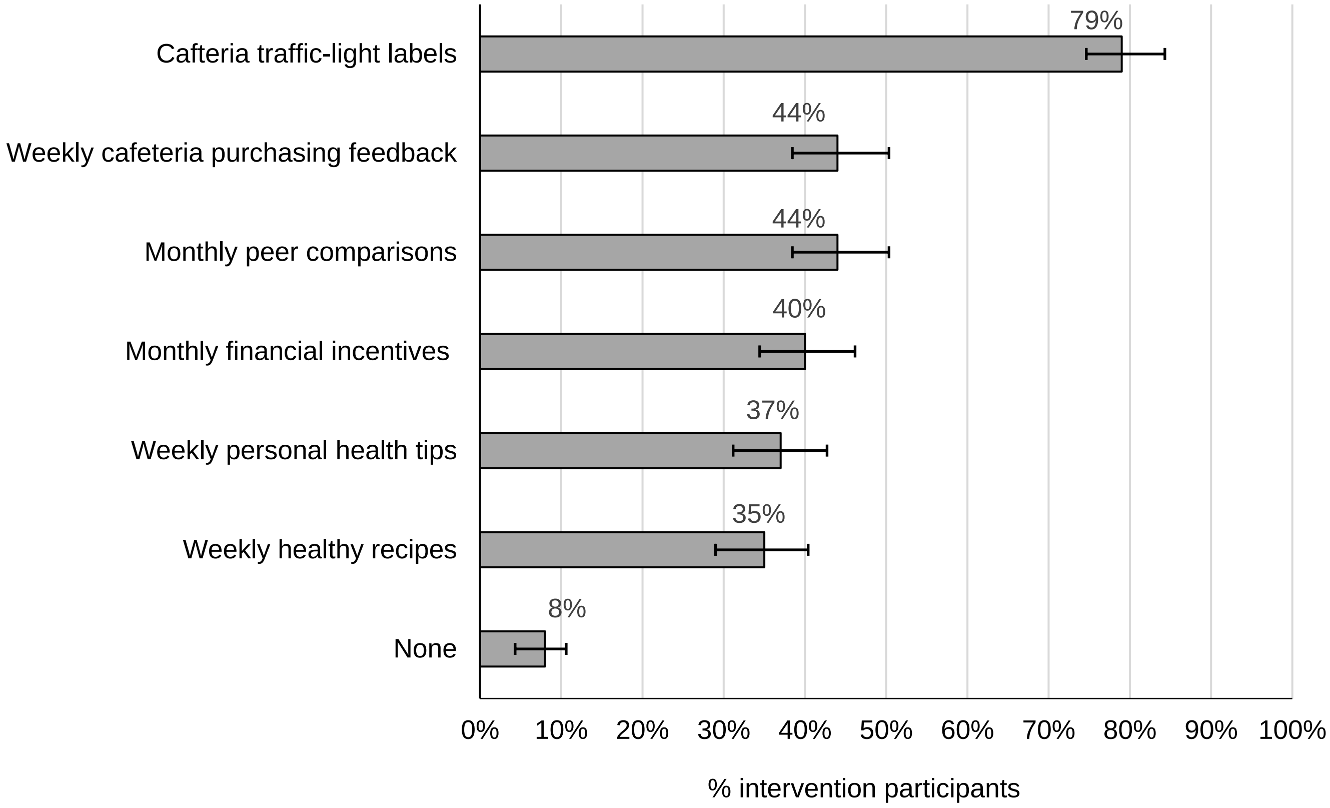

Total N=268 participants in the intervention group who completed the 12-month survey.

Error bars represent 95% Confidence Intervals.

© 2021 Thorndike AN et al. *JAMA Network Open.*

eFigure 2. Total Number of Intervention Components Rated Helpful by Intervention Participants Who Rated 1 or More Components

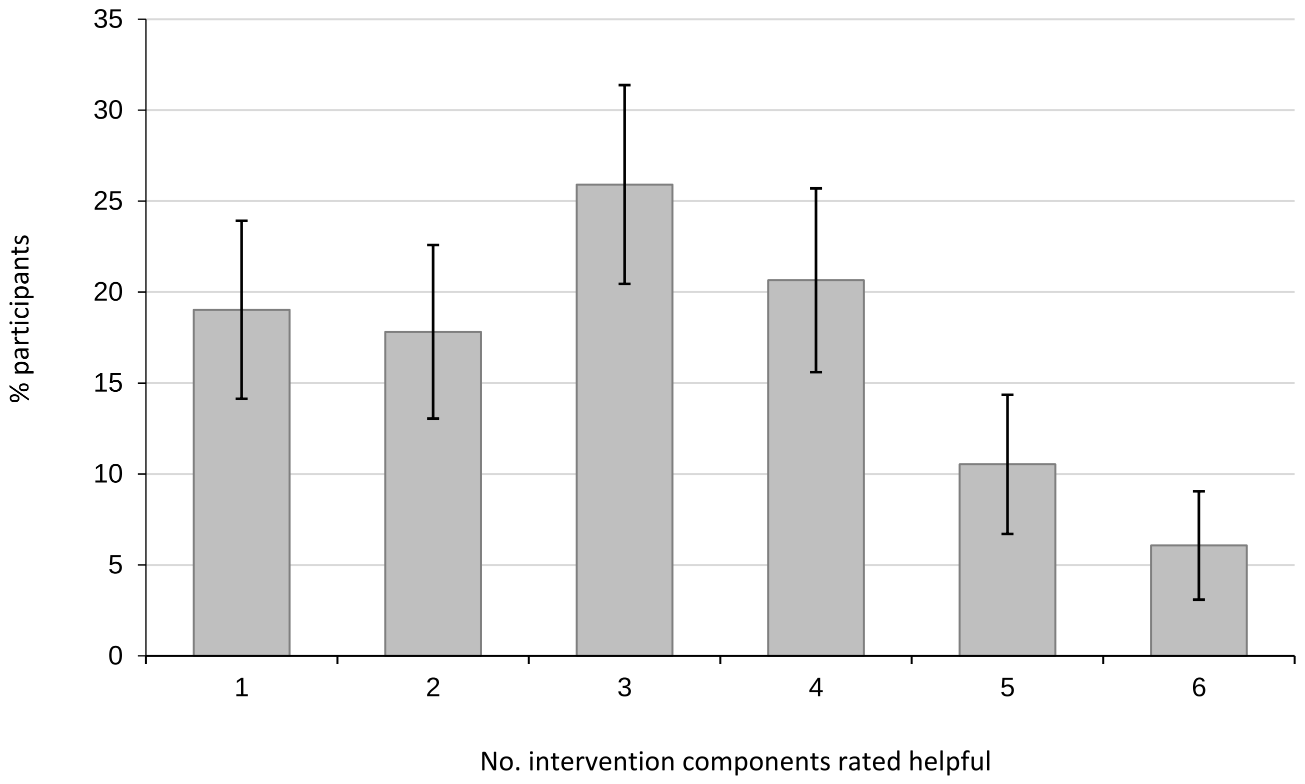

Total N=247 participants in the intervention group who completed the 12-month survey and rated one or more of the 6 intervention components as being helpful.

Error bars represent 95% Confidence Intervals.

© 2021 Thorndike AN et al. *JAMA Network Open.*
