# Supplementary material for: Automated Behavioral Workplace Intervention to Prevent Weight Gain and Improve Diet: The ChooseWell 365 Randomized Clinical Trial
Source: JAMA Netw Open. 2021 Jun 7;4(6):e2112528. doi: 10.1001/jamanetworkopen.2021.12528 (PMC8185595; doi:10.1001/jamanetworkopen.2021.12528)
Supplement: Supplement 3. — Data Sharing Statement [file jamanetwopen-e2112528-s003.pdf]

Data Sharing Statement

Thorndike. Automated Behavioral Workplace Intervention to Prevent Weight Gain and Improve Diet. *JAMA Netw Open*. Published June 07, 2021. doi:10.1001/jamanetworkopen.2021.12528

Data
Data available: No

Additional Information
Explanation for why data not available: Data cannot be shared publicly at this time because our data contains potentially identifying participant information. The study population is a relatively small cohort of employees at Massachusetts General Hospital (a named institution in the manuscript) enrolled in the ChooseWell 365 RCT during a recent time period. Sharing information on individuals' age, sex, dates, timing of cafeteria purchases, and BMI will compromise participant privacy.
